# Halogen–Metal Exchange on Bromoheterocyclics with Substituents Containing an Acidic Proton via Formation of a Magnesium Intermediate

**DOI:** 10.3390/molecules22111952

**Published:** 2017-11-11

**Authors:** Qingqiang Tian, Suqin Shang, Huajun Wang, Guoqiang Shi, Zhiyao Li, Jianyong Yuan

**Affiliations:** Pharmacy College, Chongqing Medical University, Chongqing 400016, China; Tqingqiang@163.com (Q.T.); ssqhcq@163.com (S.S.); Wanghuajun333@163.com (H.W.); 17790191007@163.com (G.S.); 18883850724@163.com (Z.L.)

**Keywords:** acidic proton, non-cryogenic, arene, bromine–metal exchange

## Abstract

A selective and practical bromine–metal exchange on bromoheterocyclics bearing substituents with an acidic proton under non-cryogenic conditions was developed by a simple modification of an existing protocol. Our protocol of using a combination of *i*-PrMgCl and *n*-BuLi has not only solved the problem of intermolecular quenching that often occurred when using alkyl lithium alone as the reagent for halogen–lithium exchange, but also offered a highly selective method for performing bromo–metal exchange on dibrominated arene compounds through chelation effect.

## 1. Introduction

**Scheme 1:**
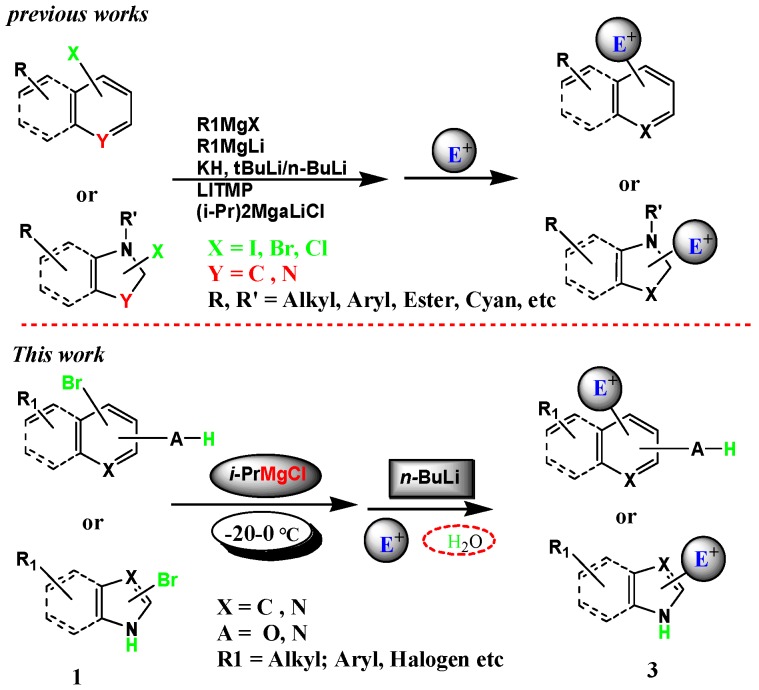
Metal halogen exchange method.

Substituted heterocyclics are an important class of heterocyclic building blocks that are routinely used in the synthesis of medicinal and agrochemical products [1]. Functionalization of heterocyclics via organometallic intermediates has been one of the most frequently used methods for the synthesis of various heterocyclic derivatives. One of the strategies relies on the rapid bromine–lithium exchange reaction on halogenated heterocyclics under cryogenic conditions at low temperature [2]. However, the reactivity and stability of the lithiated species under the reaction conditions usually require cryogenic conditions to minimize undesired side-reactions, such as nucleophilic attack of the heterocyclic ring by alkyl lithium (Scheme 1) [3,4,5,6,7,8,9,10]. The protocol reported by the literature basically allows for the lithiation of bromo-substituted heterocycles in the presence of N–H and O–H acidic protons such as indoles or phenols. Corresponding protocols are known. In these papers, as the authors did more than two equivalents of the organo-lithium reagent (or another strong base such as KH) is employed, and first deprotonation of the acidic protons takes place, followed by the lithiation [11,12,13,14]. Alternatively, the preparation of the Grinard reagent via halogen–magnesium exchange typically uses *i*-PrMgCl. The subsequent quenching with various electrophiles has been proven to be a highly useful method for the synthesis of heterocyclic derivatives [15,16,17,18,19,20,21]. In this method, the additional functional groups can tolerate the process of halogen–metal exchange, which considerably enhanced its synthetic utility. However, for those substrates that bearing an acidic proton (Compound **1**, Scheme 1), *i*-PrMgCl is generally not reactive enough to affect Br–Mg exchange. Under these circumstances, the use of more reactive alkyl lithium reagents only made limited success because of nucleophilic attack on the pyridine ring by *n*-BuLi as well as the formation of corresponding protonated products due to intermolecular quenching [22,23,24,25] (Br–Li exchange is usually fast enough to compete with the removal of an acidic proton by *n*-BuLi).

Therefore, the development of new methodologies that can address these issues is highly desirable. In this paper, we wish to report that halogen–metal exchange on heterocyclic substrates bearing an acidic proton can be performed efficiently through a combined use of *i*-PrMgCl and *n*-BuLi under essentially noncryogenic conditions and that high regioselectivity can be achieved on halogen–metal exchange on substrate with two bromo groups via chelation effect.

## 2. Results and Discussion

We started with bromopyridine **1a** that substituted with a Boc-protected amino group to explore the best condition to execute the Br–Mg exchange in the presence of an acidic carbamate proton. When Compound **1a** was treated with one to three equivalents of *i*-PrMgCl at room temperature, no expected Br–Mg exchange took place (Table 1, entries 1–3). In addition, we used three equivalents of *i*-PrMgCl, lowered the temperature to 0 °C and increased the reaction time, with no expected exchange results, resulting in a list of impurities (Table 1, entry 4). Subsequently, we used the more active lithium reagent to treat Compound **1a** with one to three equivalents of *n*-BuLi at low temperature and did not find the desired target product to obtain unwanted polymers (Table 1, entries 5–7). In this case, we considered the combination of *i*-PrMgCl and *n*-BuLi. First, we explored *i*-PrMgCl:*n*-BuLi = 1:1 equivalent and found no efficient exchange (Table 1, entry 8). Then, when we increased the amount of *i*-PrMgCl to *i*-PrMgCl:*n*-BuLi = 1:2 equivalents, compound **1a** was treated successively with one equivalent of *i*-PrMgCl at 0 °C and two equivalents of *n*-BuLi, gratifyingly a clean exchange took place and subsequent quenching of the intermediate with DMF gave a 90% yield of the expected aldehyde product **3a** (Table 1, entry 9). 

According to Knochel et al. [26], LiCl-mediated Br–Mg exchange reaction was used to prepare functionalized aryl compounds from organic bromides. Then we guessed the role of lithium in the system. We used inorganic lithium carbonate and lithium chloride instead of *n*-BuLi, and found no target product formated (Table 1, entries 10 and 11). At the same time, we used *p*-bromophenol as raw material, to further explore the role of inorganic lithium chloride in the system and found no *p*-hydroxybenzaldehyde but the starting material (Table 1, entry 12). Therefore, *n*-BuLi plays a pivotal role in the reaction.

We examined the stability of the metalated species and found that the intermediate was stable in THF at −20 °C, give **3a** with a yield of 90%, even after aging for 0.5 h before treated with tert-butyl (5-formylpyridin-2-yl) carbamate (Table 2).

**Scheme 2:**
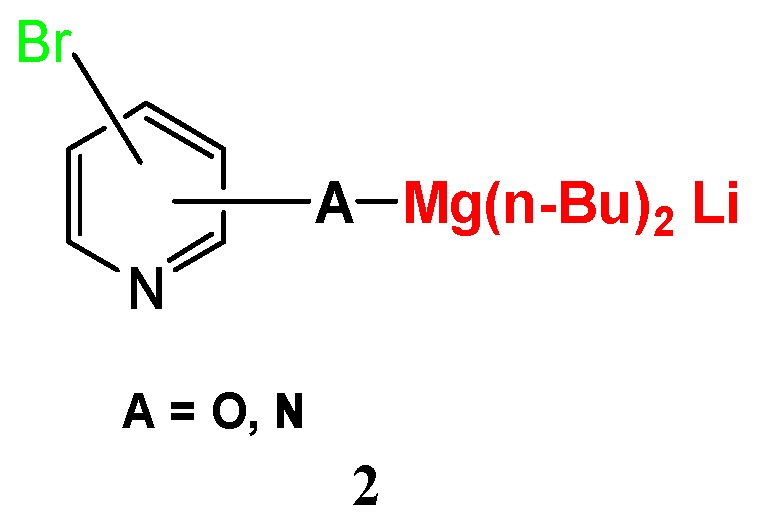
Intermediate “ate” complex.

Compared with the typical Br–Li exchange with alkyl lithium which requires to be performed under cryogenic condition, the current reaction could be performed at about 0 °C. It was found that two equivalents of *n*-BuLi were necessary for the exchange to be completed, indicating that the bromine–metal exchange was probably affected by the action of an “ate” complex (**2**) (Scheme 2) formed from one equivalent of *N*-magnesium salt and two equivalents of *n*-BuLi [27,28,29]. Similarly, following the same protocol, bromine–magnesium exchange could be performed successively on other bromopyridines bearing an acidic carbamate proton, then quenching of the organometallic intermediates with DMF and other electrophiles to afford a high yield of the corresponding products (Table 3, entry 1).

The above-mentioned efficient method for performing halogen–metal exchange on substrates bearing carbamate group(s) was established. Then, we sought to extend the same procedure to bromopyridines bearing other phenolic hydroxyl groups. We obtained the same results for the selective functionalization of the *p*-bromophenol substrate as those reported with the lithium trialkylmagnesiate complex (ref. [30,31], 56% yield). Then, we turned our attention to the effect of the acetyl amino substituent (Table 3, entry 9). The series proceeded here with the same trend as for the bromopyridines bearing an acidic carbamate proton and resulted in moderate to good yields (94%) with high selectivity.

We examined the selectivity of Br–Mg exchange on a series of dibromo-substituted aromatic hydrocarbon derivatives bearing an acidic functional group which would allow us to synthesize aromatic hydrocarbon derivatives substituted with multiple functional groups. As summarized in Table 3, when dibromo substrates **1e** and **1o** were treated with one equivalent of *i*-PrMgCl and two equivalents of *n*-BuLi, only one of the bromo groups was exclusively exchanged. Subsequent reaction of the metallated aromatic hydrocarbons with various electrophiles afforded trisubstituted aromatic hydrocarbon derivatives **3e** and **3o** with high yield (Table 3, entries 5 and 15).

To further illustrate the efficient method for bromine–metal exchange on bromopyridines and arenes bearing an acidic proton-containing substituent, we selected bromoindole and heteroaromatic substrates that have active hydrogen sensitive moieties (Table 3), such as bromoindole (entries 3, 4 and 6), bromo benzimidazole (entry 7), bromopyrrole (entry 10), bromoimidazole (entries 11 and 12). In a few cases (entries 4 and 7), the isolated yields were poor (e.g., <80%), which can be attributed to a combination of steric and electronic factors or to the volatility of the product (entries 4 and 7).

**Scheme 3:**
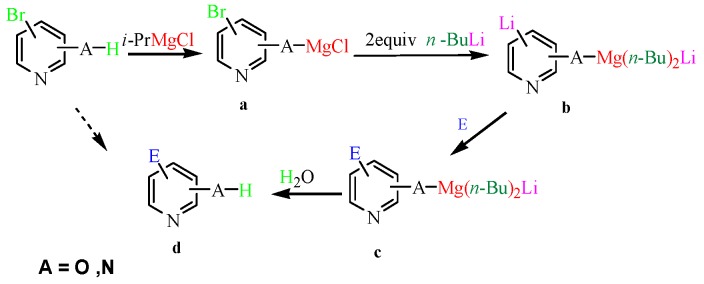
Proposed mechanism.

Based on the above results and the published literature [32,33,34,35], we postulated a tentative mechanism of halogen–metal exchange in Scheme 3. Initially, we used one equiv. of *i*-PrMgCl to remove the active proton hydrogen from the aromatic hydrocarbon at 0 °C to form the organomagnesium reagent (**a**), and then added two equivs. of *n*-BuLi at −20 °C. A half of the *n*-BuLi combined with the magnesium salt to form an “ate” complex and the rest of n-BuLi exchanged with the halogen to form magnesium compound (**b**). Next, one equiv. of an electrophilic reagent was coupled to the C–Li bond (**c**). Finally, water was added to provide proton hydrogen to obtain the desired product (**d**).

## 3. Materials and Methods 

General remarks: All reagents were obtained from Aladdin Reagent Shanghai Co., Ltd. (Shanghai, China), Lagewell Technology Co., Ltd., Meyer Reagent Shanghai Co., Ltd., Macklin Reagent Shanghai Co., Ltd., Chongqing Chuandong Chemical Co., Ltd. etc. without further purification unless otherwise noted. High resolution mass spectra (South China Agricultural University, Guangzhou) were measured on commercial instruments. NMR spectra were recorded on commercial instruments (Bruker company, Karlsruhe, Germany) and operated at 600 MHz for ^1^H-NMR and 151 MHz for ^13^C-NMR. Chemical shifts were reported in ppm from tetramethylsilane with the solvent resonance as the internal standard ((CD_3_)_2_SO, δ = 2.50, δ = 3.33) in ^1^H-NMR spectra and chemical shifts were reported in ppm from the tetramethylsilane with the solvent resonance as internal standard ((CD_3_)_2_SO, δ = 39.5) in ^13^C-NMR spectra. Spectra are reported as follows: chemical shift (δ ppm), multiplicity (s = singlet, d = doublet, t = triplet, q = quartet, m = multiplet), coupling constants (Hz), integration and assignment.

*(5-Formyl-pyridin-2-yl)-carbamic acid tert-butyl ester* (**3a**): To a solution of (5-Bromo-pyridin-2-yl)-carbamic acid tert-butyl ester (1.0 g, 3.7 mmol, 1.0 equiv.) in dry THF (12 mL) at 0 °C was added a 2 M solution of *i*-PrMgCl in THF (1.85 mL, 3.7 mmol, 1.0 equiv.) during 5 min. The clear solution was stirred at that temperature for an additional 5 min, and a 2.5 M solution of *n*-BuLi in hexanes (3 mL, 7.5 mmol, 2.0 equiv.) was added dropwise during 5 min, while maintaining the temperature below −20 °C. The resulting mixture was stirred at that temperature for 0.5 h, dry DMF (0.27 g, 3.7 mmol, 1.0 equiv.) in dry THF (5 mL) was added dropwise during 10 min. The resulting mixture was warmed to −20 °C in 0.5 h and quenched with water (6 mL). After stirring the mixture below −20 °C for 10 min, the phases were separated and the water phase was extracted one additional time with ethyl acetate. The resulting suspension was allowed to reach room temperature and fitered through a 0.5 × 1 cm pad of silica gel eluted with 10 mL of ethyl acetate. The filtrate was concentrated and the residue was purified by flash chromatography on silica gel (eluent: petroleum ether/ethyl acetate = 10:1) to afford product **3a** as white solid, 0.73 g (yield: 90%). ^1^H-NMR (600 MHz, DMSO) δ 10.42 (s, 1H), 9.94 (s, 1H), 8.93–8.60 (m, 1H), 8.17 (dd, *J* = 8.8, 2.3 Hz, 1H), 7.99 (d, *J* = 8.8 Hz, 1H), 1.48 (s, 9H). ^13^C-NMR (151 MHz, DMSO) δ 191.18, 157.08, 152.82, 152.40, 138.23, 127.30, 112.23, 80.97, 28.37.

*1H-Indole-2-carboxylic acid* (**3b**): To a solution of 2-Bromo-1*H*-indole (1.0 g, 5 mmol, 1.0 equiv.) in dry THF (20 mL) at 0 °C was added a 2 M solution of *i*-PrMgCl in THF (2.5 mL, 5 mmol, 1.0 equiv.) during 5 min. The clear solution was stirred at that temperature for an additional 5 min, and a 2.5 M solution of nBuLi in hexanes (4 mL, 10 mmol, 2.0 equiv.) was added dropwise during 5 min, while maintaining the temperature below −20 °C. The resulting mixture was stirred at that temperature for 0.5 h, dry CO_2_ (0.22 g, 5 mmol, 1.0 equiv.) was added to −20 °C. The resulting mixture was warmed to −20 °C in 0.5 h and quenched with water (6 mL). After stirring the mixture below −20 °C for 10 min, the phases were separated and the water phase was extracted one additional time with ethyl acetate. The resulting suspension was allowed to reach room temperature and fitered through a 0.5 × 1 cm pad of silica gel eluted with 10 mL of ethyl acetate. The filtrate was concentrated and the residue was purified by flash chromatography on silica gel (eluent: petroleum ether/ethyl acetate = 3:1) to afford product **3b** as white solid, 0.7 g (yield: 85%), m.p.: 203–204 °C. ^1^H-NMR (600 MHz, DMSO) δ 12.94 (s, 1H), 11.76 (s, 1H), 7.79–6.89 (m, 5H). ^13^C-NMR (151 MHz, DMSO) δ 163.29, 137.71, 128.88, 127.34, 124.73, 122.40, 120.41, 112.95, 107.77.

*1H-Indole-3-carboxylic acid* (**3c**): To a solution of 3-Bromo-1*H*-indole (0.86 g, 4.4 mmol, 1.0 equiv.) in dry THF (20 mL) at 0 °C was added a 2 M solution of *i*-PrMgCl in THF (2.2 mL, 4.4 mmol 1.0 equiv.) during 5 min. The clear solution was stirred at that temperature for an additional 5 min, and a 2.5 M solution of *n*-BuLi in hexanes (3.5 mL, 8.8 mmol, 2.0 equiv.) was added dropwise during 5 min, while maintaining the temperature below −20 °C. The resulting mixture was stirred at that temperature for 0.5 h, dry CO_2_ (0.2 g, 4.4 mmol, 1.0 equiv.) was added to −20 °C. The resulting mixture was warmed to −20 °C in 0.5 h and quenched with water (6 mL). After stirring the mixture below −20 °C for 10 min, the phases were separated and the water phase was extracted one additional time with ethyl acetate. The resulting suspension was allowed to reach room temperature and fitered through a 0.5 × 1 cm pad of silica gel eluted with 10 mL of ethyl acetate. The filtrate was concentrated and the residue was purified by flash chromatography on silica gel (eluent: petroleum ether/ethyl acetate = 3:1) to afford product **3c** as off-white solid, 0.63 g (yield: 89%), m.p.: 193–196 °C. ^1^H-NMR (600 MHz, DMSO) δ 11.82 (s, 1H), 8.02 (t, *J* = 5.7 Hz, 2H), 7.47 (d, *J* = 7.6 Hz, 1H), 7.32–6.99 (m, 2H). ^13^C-NMR (151 MHz, DMSO) δ 166.43, 136.89, 132.71, 126.48, 122.58, 121.42, 121.05, 112.65, 107.87.

*1H-Indole-5-carboxylic acid* (**3d**): To a solution of 5-Bromo-1*H*-indole (0.86 g, 4.4 mmol, 1.0 equiv.) in dry THF (20 mL) at 0 °C was added a 2 M solution of *i*-PrMgCl in THF (2.2 mL, 4.4 mmol, 1 equiv.) during 5 min. The clear solution was stirred at that temperature for an additional 5 min, and a 2.5 M solution of *n*-BuLi in hexanes (3.5 mL, 8.8 mmol, 2.0 equiv.) was added dropwise during 5 min, while maintaining the temperature below −20 °C. The resulting mixture was stirred at that temperature for 0.5 h, dry CO_2_ (0.2 g, 4.4 mmol, 1.0 equiv.) was added to −20 °C. The resulting mixture was warmed to −20 °C in 0.5 h and quenched with water (6 mL). After stirring the mixture below −20 °C for 10 min, the phases were separated and the water phase was extracted one additional time with ethyl acetate. The resulting suspension was allowed to reach room temperature and fitered through a 0.5 × 1 cm pad of silica gel eluted with 10 mL of ethyl acetate. The filtrate was concentrated and the residue was purified by flash chromatography on silica gel (eluent: petroleum ether/ethyl acetate = 3:1) to afford product **3d** as off-white solid, 0.46 g (yield: 65%), m.p.: 210–214 °C. ^1^H-NMR (600 MHz, DMSO) δ 12.39 (s, 1H), 11.46 (s, 1H), 8.25 (s, 1H), 7.72 (dd, *J* = 8.5, 1.5 Hz, 1H), 7.45 (dd, *J* = 8.4, 5.7 Hz, 2H), 6.57 (s, 1H). ^13^C-NMR (151 MHz, DMSO) δ 168.90, 138.80, 127.64, 127.35, 123.28, 122.67, 121.87, 111.57, 102.93.

*5-Bromo-1H-indole-3-carbaldehyde* (**3e**): To a solution of 3,5-dibromo-1*H*-indole (1.2 g, 4.4 mmol, 1.0 equiv.) in dry THF (20 mL) at 0 °C was added a 2 M solution of iPrMgCl in THF (2.2 mL, 4.4 mmol, 1.0 equiv.) during 5 min. The clear solution was stirred at that temperature for an additional 5 min, and a 2.5 M solution of *n*-BuLi in hexanes (3.5 mL, 8.8 mmol, 2.0 equiv.) was added dropwise during 5 min, while maintaining the temperature below −20 °C. The resulting mixture was stirred at that temperature for 0.5 h, dry DMF (0.32 g, 4.4 mmol, 1.0 equiv.) was added to −20 °C. The resulting mixture was warmed to −20 °C in 0.5 h and quenched with water (6 mL). After stirring the mixture below −20 °C for 10 min, the phases were separated and the water phase was extracted one additional time with ethyl acetate. The resulting suspension was allowed to reach room temperature and fitered through a 0.5 × 1 cm pad of silica gel eluted with 10 mL of ethyl acetate. The filtrate was concentrated and the residue was purified by flash chromatography on silica gel (eluent: petroleum ether/ethyl acetate = 3:1) to afford product **3e** as yellow solid, 0.78 g (yield: 80%), m.p.: 192–194 °C. ^1^H-NMR (600 MHz, DMSO) δ 12.35 (s, 1H), 9.93 (s, 1H), 8.47–8.05 (m, 2H), 7.57–7.21 (m, 2H). ^13^C-NMR (151 MHz, DMSO) δ 185.57, 139.67, 136.23, 126.49, 126.36, 123.39, 117.90, 115.27, 115.01.

*5-Methoxy-1H-indole-2-carboxylic acid* (**3f**): To a solution of 2-Bromo-5-methoxy-1*H*-indole (1.0 g, 4.4 mmol, 1.0 equiv.) in dry THF (20 mL) at 0 °C was added a 2 M solution of *i*-PrMgCl in THF (2.2 mL, 4.4 mmol, 1.0 equiv.) during 5 min. The clear solution was stirred at that temperature for an additional 5 min, and a 2.5 M solution of *n*-BuLi in hexanes (3.5 mL, 8.8 mmol, 2.0 equiv.) was added dropwise during 5 min, while maintaining the temperature below −20 °C. The resulting mixture was stirred at that temperature for 0.5 h, dry CO_2_ (0.20 g, 1.0 equiv.) was added to −20 °C. The resulting mixture was warmed to −20 °C in 0.5 h and quenched with water (6 mL). After stirring the mixture below −20 °C for 10 min, the phases were separated and the water phase was extracted one additional time with ethyl acetate. The resulting suspension was allowed to reach room temperature and fitered through a 0.5 × 1 cm pad of silica gel eluted with 10 mL of ethyl acetate. The filtrate was concentrated and the residue was purified by flash chromatography on silica gel (eluent: petroleum ether/ethyl acetate = 3:1) to afford product **3f** as brown solid, 0.68 g (yield: 80%), m.p.: 199–201 °C. ^1^H-NMR (600 MHz, DMSO) δ 7.36 (d, *J* = 8.9 Hz, 1H), 7.06 (d, *J* = 23.6 Hz, 2H), 6.90 (d, *J* = 8.8 Hz, 1H), 3.73 (s, 3H). ^13^C-NMR (151 MHz, DMSO) δ 163.25, 154.31, 133.07, 129.10, 127.65, 116.28, 113.83, 107.47, 102.44, 55.61.

*1H-Benzoimidazole-5-carboxylic acid* (**3g**): To a solution of 5-Bromo-1*H*-benzimidazole (0.87 g, 4.4 mmol, 1.0 equiv.) in dry THF (20 mL) at 0 °C was added a 2 M solution of *i*-PrMgCl in THF (2.2 mL, 1 equiv.) during 5 min. The clear solution was stirred at that temperature for an additional 5 min, and a 2.5 M solution of *n*-BuLi in hexanes (3.5 mL, 8.8 mmol, 2.0 equiv.) was added dropwise during 5 min, while maintaining the temperature below −20 °C. The resulting mixture was stirred at that temperature for 0.5 h, dry CO_2_ (0.20 g, 4.4mmol, 1.0 equiv.) was added to −20 °C. The resulting mixture was warmed to −20 °C in 0.5 h and quenched with water (6 mL). After stirring the mixture below −20 °C for 10 min, the phases were separated and the water phase was extracted one additional time with ethyl acetate. The resulting suspension was allowed to reach room temperature and fitered through a 0.5 × 1 cm pad of silica gel eluted with 10 mL of ethyl acetate. The filtrate was concentrated and the residue was purified by flash chromatography on silica gel (eluent:petroleum ether/ethyl acetate = 3:1) to afford product **3g** as brown solid, 0.5 g (yield: 71%). ^1^H-NMR (600 MHz, DMSO) δ 8.43 (s, 1H), 8.26 (s, 1H), 7.87 (d, *J* = 8.4 Hz, 1H), 7.67 (d, *J* = 8.4 Hz, 1H). ^13^C-NMR (151 MHz, DMSO) δ 168.47, 144.83, 125.10, 123.74, 118.13, 115.12.

*4-Hydroxy-benzaldehyde* (**3h**): To a solution of 4-Bromo-phenol (1.5 g, 8.7 mmol, 1 equiv.) in dry THF (25 mL) at 0 °C was added a 2 M solution of *i*-PrMgCl in THF (4.3 mL, 8.7 mmol, 1.0 equiv.) during 5 min. The clear solution was stirred at that temperature for an additional 5 min, and a 2.5 M solution of *n*-BuLi in hexanes (7.0 mL, 17.4 mmol, 2.0 equiv.) was added dropwise during 5 min, while maintaining the temperature below −20 °C. The resulting mixture was stirred at that temperature for 0.5 h, dry DMF (0.63 g, 8.7 mmol, 1.0 equiv.) in dry THF (5 mL) was added dropwise during 10 min. The resulting mixture was warmed to −20 °C in 0.5 h and quenched with water (6 mL). After stirring the mixture below −20 °C for 10 min, the phases were separated and the water phase was extracted one additional time with ethyl acetate. The resulting suspension was allowed to reach room temperature and fitered through a 0.5 × 1 cm pad of silica gel eluted with 10 mL of ethyl acetate. The filtrate was concentrated and the residue was purified by flash chromatography on silica gel (eluent:petroleum ether/ethyl acetate = 5:1) to afford product **3h** as white solid, 0.94 g (yield: 90%), m.p.: 114–116 °C. ^1^H-NMR (600 MHz, DMSO) δ 10.59 (s, 1H), 9.78 (s, 1H), 7.75 (d, *J* = 8.6 Hz, 2H), 6.93 (d, *J* = 8.6 Hz, 2H). ^13^C-NMR (151 MHz, DMSO) δ 191.35, 191.33, 163.76, 132.53, 128.89, 116.29.

*N-(4-Formyl-phenyl)-acetamide* (**3i**): To a solution of 4-Bromo-phen *N*-(4-Formyl -phenyl)-acetamide (1.5 g, 7.0 mmol, 1.0 equiv.) in dry THF (25 mL) at 0 °C was added a 2 M solution of iPrMgCl in THF (3.5 mL, 7.0 mmol, 1.0 equiv.) during 5 min. The clear solution was stirred at that temperature for an additional 5 min, and a 2.5 M solution of *n*-BuLi in hexanes (5.6 mL, 14.0 mmol, 2.0 equiv.) was added dropwise during 5 min, while maintaining the temperature below −20 °C. The resulting mixture was stirred at that temperature for 0.5 h, dry DMF (0.5 g, 1.0 equiv.) in dry THF (5 mL) was added dropwise during 10 min. The resulting mixture was warmed to −20 °C in 0.5 h and quenched with water (6 mL). After stirring the mixture below −20 °C for 10 min, the phases were separated and the water phase was extracted one additional time with ethyl acetate. The resulting suspension was allowed to reach room temperature and fitered through a 0.5 × 1 cm pad of silica gel eluted with 10 mL of ethyl acetate. The filtrate was concentrated and the residue was purified by flash chromatography on silica gel (eluent: petroleum ether/ethyl acetate = 10:1) to afford product **3i** as white solid, 1.0 g (yield: 94%) , m.p.: 157–158 °C. ^1^H-NMR (600 MHz, DMSO) δ 10.35 (s, 1H), 9.86 (s, 1H), 7.81 (dd, *J* = 32.9, 8.6 Hz, 4H), 2.10 (s, 3H). ^13^C-NMR (151 MHz, DMSO) δ 191.92, 169.55, 145.29, 131.56, 131.27, 119.01, 24.68.

*1H-Pyrrole-2-carbaldehyde* (**3j**): To a solution of 2-Bromo-1*H*-pyrrole (0.5 g, 3.4 mmol, 1.0 equiv.) in dry THF (15 mL) at 0 °C was added a 2 M solution of *i*-PrMgCl in THF (1.7 mL, 3.4 mmol, 1.0 equiv.) during 5 min. The clear solution was stirred at that temperature for an additional 5 min, and a 2.5 M solution of *n*-BuLi in hexanes (2.7 mL, 6.8 mmol, 2.0 equiv.) was added dropwise during 5 min, while maintaining the temperature below −20 °C. The resulting mixture was stirred at that temperature for 0.5 h, dry DMF (0.25 g, 1.0 equiv.) in dry THF (5 mL) was added dropwise during 10 min. The resulting mixture was warmed to −20 °C in 0.5 h and quenched with water (6 mL). After stirring the mixture below −20 °C for 10 min, the phases were separated and the water phase was extracted one additional time with ethyl acetate. The resulting suspension was allowed to reach room temperature and fitered through a 0.5 × 1 cm pad of silica gel eluted with 10 mL of ethyl acetate. The filtrate was concentrated and the residue was purified by flash chromatography on silica gel (eluent:petroleum ether/ethyl acetate = 10:1) to afford product **3j** as white solid, 0.29 g (yield: 89%) , m.p.: 42–44 °C. ^1^H-NMR (600 MHz, DMSO) δ 12.19 (s, 1H), 11.69 (s, 1H), 7.04–6.85 (m, 1H), 6.72 (dd, *J* = 4.2, 2.9 Hz, 1H), 6.12 (dd, *J* = 5.8, 2.4 Hz, 1H). ^13^C-NMR (151 MHz, DMSO) δ 162.31, 123.82, 123.35, 115.11, 109.73.

*1H-Imidazole-2-carbaldehyde* (**3k**): To a solution of 2-bromo-1H-imidazole (0.65 g, 4.4 mmol, 1.0 equiv.) in dry THF (20 mL) at 0 °C was added a 2 M solution of *i*-PrMgCl in THF (2.2 mL, 4.4 mmol, 1.0 equiv.) during 5 min. The clear solution was stirred at that temperature for an additional 5 min, and a 2.5 M solution of *n*-BuLi in hexanes (3.5 mL, 8.8 mmol, 2.0 equiv.) was added dropwise during 5 min, while maintaining the temperature below −20 °C. The resulting mixture was stirred at that temperature for 0.5 h, dry DMF (0.32 g, 4.4 mmol, 1.0 equiv.) was added to −20 °C. The resulting mixture was warmed to −20 °C in 0.5 h and quenched with water (6 mL). After stirring the mixture below −20 °C for 10 min, the phases were separated and the water phase was extracted one additional time with ethyl acetate. The resulting suspension was allowed to reach room temperature and fitered through a 0.5 × 1cm pad of silica gel eluted with 10 mL of ethyl acetate. The filtrate was concentrated and the residue was purified by flash chromatography on silica gel (eluent: petroleum ether/ethyl acetate = 10:1) to afford product **3k** as pale yellow solid, 0.38 g (yield: 91%), m.p.: 205–206 °C. ^1^H-NMR (600 MHz, DMSO) δ 13.60 (s, 1H), 9.64 (s, 1H), 7.42 (s, 2H). ^13^C-NMR (151 MHz, DMSO) δ 181.66, 146.09.

*1H-Imidazole-4-carbaldehyde* (**3l**): To a solution of 4-bromo-1*H*-imidazole (0.65 g, 4.4 mmol, 1.0 equiv.) in dry THF (20 mL) at 0 °C was added a 2 M solution of *i*-PrMgCl in THF (2.2 mL, 4.4 mmol, 1.0 equiv.) during 5 min. The clear solution was stirred at that temperature for an additional 5 min, and a 2.5 M solution of *n*-BuLi in hexanes (3.5 mL, 8.8 mmol, 2.0 equiv.) was added dropwise during 5 min, while maintaining the temperature below −20 °C. The resulting mixture was stirred at that temperature for 0.5 h, dry DMF (0.32 g, 4.4 mmol, 1.0 equiv.) was added to −20 °C. The resulting mixture was warmed to −20 °C in 0.5 h and quenched with water (6 mL). After stirring the mixture below −20 °C for 10 min, the phases were separated and the water phase was extracted one additional time with ethyl acetate. The resulting suspension was allowed to reach room temperature and fitered through a 0.5 × 1 cm pad of silica gel eluted with 10 mL of ethyl acetate. The filtrate was concentrated and the residue was purified by flash chromatography on silica gel (eluent:petroleum ether/ethyl acetate = 10:1) to afford product **3l** as off-white solid, 0.36 g (yield: 85%), m.p.: 175–177 °C. ^1^H-NMR (600 MHz, DMSO) δ 9.74 (s, 1H), 7.99 (s, 1H), 7.94 (s, 1H). ^13^C-NMR (151 MHz, DMSO) δ 184.46, 139.44, 134.9, 129.5.

*6-Hydroxy-pyridine-2-carboxylic acid* (**3m**): To a solution of 2-bromo-6-hydroxypyridine (0.76 g, 4.4 mmol, 1.0 equiv.) in dry THF (20 mL) at 0 °C was added a 2 M solution of -PrMgCl in THF (2.2 mL, 4.4 mmol, 1.0 equiv.) during 5 min. The clear solution was stirred at that temperature for an additional 5 min, and a 2.5 M solution of *n*-BuLi in hexanes (3.5 mL, 8.8 mmol, 2.0 equiv.) was added dropwise during 5min, while maintaining the temperature below −20 °C. The resulting mixture was stirred at that temperature for 0.5 h, dry CO_2_ (0.20 g, 1.0 equiv.) was added to −20 °C. The resulting mixture was warmed to −20 °C in 0.5 h and quenched with water (6 mL). After stirring the mixture below −20 °C for 10 min, the phases were separated and the water phase was extracted one additional time with ethyl acetate. The resulting suspension was allowed to reach room temperature and fitered through a 0.5 × 1 cm pad of silica gel eluted with 10 mL of ethyl acetate. The filtrate was concentrated and the residue was purified by flash chromatography on silica gel (eluent: petroleum ether/ethyl acetate = 10:1) to afford product **3m** as off-white solid, 0.56 g (yield: 93%) , m.p.: 275–277 °C. ^1^H-NMR (600 MHz, DMSO) δ 7.56 (dd, *J* = 8.9, 7.0 Hz, 1H), 6.97 (d, *J* = 6.8 Hz, 1H), 6.65 (d, *J* = 9.0 Hz, 1H). ^13^C-NMR (151 MHz, DMSO) δ 163.28, 162.67, 140.51, 137.97, 123.88, 110.42.

*2-Hydroxy-nicotinic acid* (**3n**): To a solution of 3-bromo-2-hydroxypyridine (0.76 g, 4.4 mmol, 1.0 equiv.) in dry THF (20 mL) at 0 °C was added a 2 M solution of *i*-PrMgCl in THF (2.2 mL, 4.4 mmol, 1.0 equiv.) during 5 min. The clear solution was stirred at that temperature for an additional 5 min, and a 2.5 M solution of *n*-BuLi in hexanes (3.5 mL, 8.8 mmol, 2.0 equiv.) was added dropwise during 5 min, while maintaining the temperature below −20 °C. The resulting mixture was stirred at that temperature for 0.5 h, dry CO_2_ (0.20 g, 1.0 equiv.) was added to −20 °C. The resulting mixture was warmed to −20 °C in 0.5 h and quenched with water (6 mL). After stirring the mixture below −20 °C for 10 min, the phases were separated and the water phase was extracted one additional time with ethyl acetate. The resulting suspension was allowed to reach room temperature and fitered through a 0.5 × 1 cm pad of silica gel eluted with 10 mL of ethyl acetate. The filtrate was concentrated and the residue was purified by flash chromatography on silica gel (eluent: petroleum ether/ethyl acetate = 10:1) to afford product **3n** as off-white solid, 0.48 g (yield: 79%), m.p.: 255–257 °C. ^1^H-NMR (600 MHz, DMSO) δ 14.76 (s, 1H), 13.38 (s, 1H), 8.38 (dd, *J* = 7.2, 2.0 Hz, 1H), 7.95 (dd, *J* = 6.3, 2.0 Hz, 1H), 6.68 (t, *J* = 6.7 Hz, 1H). ^13^C-NMR (151 MHz, DMSO) δ 165.46, 165.04, 146.61, 141.95, 117.12, 109.09.

*5-Bromo-3-hydroxyethynyl-pyridin-2-ol* (**3o**): To a solution of 3,5-dibromo-2-hydroxypyridine (1.1 g, 4.4 mmol, 1.0 equiv.) in dry THF (20 mL) at 0 °C was added a 2 M solution of *i*-PrMgCl in THF (2.2 mL, 4.4 mmol, 1.0 equiv.) during 5 min. The clear solution was stirred at that temperature for an additional 5 min, and a 2.5 M solution of *n*-BuLi in hexanes (3.5 mL, 8.8 mmol, 2.0 equiv.) was added dropwise during 5 min, while maintaining the temperature below −20 °C. The resulting mixture was stirred at that temperature for 0.5 h, dry CO_2_ (0.20 g, 4.4 mmol, 1.0 equiv.) was added to −20 °C. The resulting mixture was warmed to −20 °C in 0.5 h and quenched with water (6 mL). After stirring the mixture below −20 °C for 10 min, the phases were separated and the water phase was extracted one additional time with ethyl acetate. The resulting suspension was allowed to reach room temperature and fitered through a 0.5 × 1 cm pad of silica gel eluted with 10 mL of ethyl acetate. The filtrate was concentrated and the residue was purified by flash chromatography on silica gel (eluent: petroleum ether/ethyl acetate = 10:1) to afford product **3o** as Off-white solid, 1.0 g (yield: 80%), m.p.: 245–247 °C. ^1^H-NMR (600 MHz, DMSO) δ 13.82 (s, 2H), 8.31 (d, *J* = 2.8 Hz, 1H), 8.23 (d, *J* = 2.8 Hz, 1H). ^13^C-NMR (151 MHz, DMSO) δ 164.43, 163.72, 147.95, 142.51, 118.49, 99.96.

Experimental procedures and analytical data of all compounds (^1^H-and ^13^C-NMR), copy of the ^1^H, ^13^C and data are available in the Supplementary Materials. 

## 4. Conclusions

In summary, we have developed an efficient method for performing bromine–metal exchange process under non-cryogenic conditions on bromopyridines and arenes bearing an acidic proton-containing substituent. The use of a combination of *i*-PrMgCl and *n*-BuLi in this method has solved the problem of intermolecular quenching that is often encountered when using alkyl lithium. Moreover, the method is particularly suitable for aryl bromides of poor reactivity.

## Figures and Tables

**Table 1:**
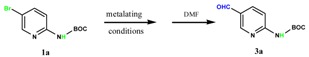
Tert-butyl (5-formylpyridin-2-yl) carbamate of metalating reagents prepared from 2-(*N*-BOC-amino)-5-bromopyridine.

Entry	Metalating Reagent	Reaction Conditions	Yield ^a^ of 3a (%)
1	*i*-PrMgCl (1.0 equiv.)	THF, rt, 24 h	Trace
2	*i*-PrMgCl (2.0 equiv.)	THF, rt, 24 h	Trace
3	*i*-PrMgCl (3.0 equiv.)	THF, rt, 24 h	Trace
4	*i*-PrMgCl (3.0 equiv.)	THF, 0 °C, 36 h	Trace
5	*n*-BuLi (1.0 equiv.)	THF, −78 °C, 24 h	Trace
6	*n*-BuLi (2.0 equiv.)	THF, −20 °C, 0.5 h	Trace
7	*n*-BuLi (2.0 equiv.)	THF, −20 °C, 0.5 h	Trace
8 ^b^	*i*-PrMgCl + *n*-BuLi (1.0:1.0 equiv.)	THF, −20 °C, 0.5 h	Trace
9 ^c^	*i*-PrMgCl + *n*-BuLi (1.0:2.0 equiv.)	THF, −20 °C, 0.5 h	90
10	*i*-PrMgCl + Li_2_CO_3_ (1.0:2.0 equiv.)	THF, −20 °C, 0.5 h	Trace
11	*i*-PrMgCl + LiCl (1.0:2.0 equiv.)	THF, −20 °C, 0.5 h	Trace
12 ^d^	*i*-PrMgCl + LiCl (1.0:2.0 equiv.)	THF, −20 °C, 0.5 h	Trace ^e^

^a^ Yields were determined by HPLC; ^b^ reaction conditions: *i*-PrMgCl (1.0 equiv.), THF, at 0 °C, then *n*-BuLi (2.0 equiv.) at −20 °C; ^c^ reaction conditions: *i*-PrMgCl (1.0 equiv.), THF, at 0 °C, then *n*-BuLi (2.0 equiv.) at −20 °C; ^d^ raw materials for bromophenol; ^e^ starting material only.

**Table 2:**
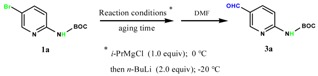
Stability of metalating reagents prepared from 2-(*N*-BOC-amino)-5-bromopyridine.

Aging Time (h)	Yield ^a^
0.25	69
0.5	90
1	75
2	40
5	23

^a^ Yields were determined by HPLC analysis.

**Table 3:** Br–Mg (Li) exchange on bromopyridines bearing an acidic proton ^a^.

Entry	Substrate	Electrophile	Product	Yield ^b^, %
1	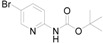 **1a**	DMF	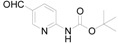 **3a**	90
2	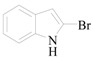 **1b**	CO_2_	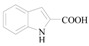 **3b**	85
3	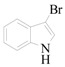 **1c**	CO_2_	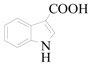 **3c**	89
4	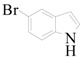 **1d**	CO_2_	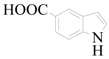 **3d**	65
5	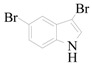 **1e**	DMF	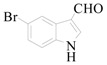 **3e**	80
6	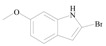 **1f**	CO_2_	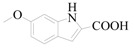 **3f**	80
7	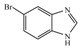 **1g**	CO_2_	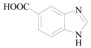 **3g**	71
8	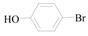 **1h**	DMF	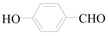 **3h**	90
9	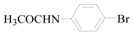 **1i**	DMF	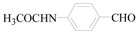 **3i**	94
10	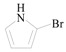 **1j**	DMF	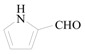 **3j**	89
11	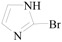 **1k**	DMF	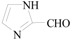 **3k**	91
12	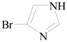 **1l**	DMF	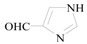 **3l**	85
13	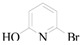 **1m**	CO_2_	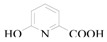 **3m**	93
14	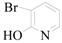 **1n**	CO_2_	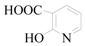 **3n**	79
15	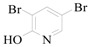 **1o**	CO_2_	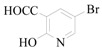 **3o**	80

^a^ Reaction conditions: *i*-PrMgCl (1.0 equiv.), THF, 0 °C, then *n*-BuLi (2.0 equiv.) −20 °C; ^b^ yield of isolated product.

## Supplementary Materials

The following are available online, general procedure for Halogen-metal exchange reaction and ^1^H and ^13^C NMR spectra of all products.
