# Supplementary material for: Halogen–Metal Exchange on Bromoheterocyclics with Substituents Containing an Acidic Proton via Formation of a Magnesium Intermediate
Source: Molecules. 2017 Nov 11;22(11):1952. doi: 10.3390/molecules22111952 (PMC6150384; doi:10.3390/molecules22111952)
Supplement: Supplementary file 1 [file molecules-22-01952-s001.pdf]

Supporting Information for

Halogen-metal Exchange on Bromoheterocyclics with Substituents Containing an Acidic Proton via Formation of a Magnesium Intermediate

Qingqiang Tian,[a] Suqin Shang,[a] Huajun Wang,[a] Guoqiang Shi,[a] Zhiyao Li,[a] Jianyong Yuan*[a]

Pharmacy College,Chongqing Medical University, Chongqing 400016,China;

E-Mail:**Tqingqiang@163.com(QQ)**; **Wanghuajun333@163.com(HJ)**; **ssqhcq@163.com(SQ)**; **17790191007@163.com(GQ)**; **18883850724@163.com(ZY)**; **enediyne@163.com(JY)**

* Author to whom correspondence should be addressed; E-Mail: enediyne@163.com(JY); Tel./Fax: +86-23-6848-5161;

Academic Editor: xxx

Received:18 October 2017; Accepted: 09 November 2017; Published: date

Table of Contents

A. General information 2
B. General Procedure for Halogen-metal Exchange Reaction 2
C. Copies of ^{1}H and ^{13}C NMR spectra of all products. 19
D. References 34

A. General information

General remarks: All reagents were obtained from Aladdin Reagent Shanghai Co., Ltd., Lagewell Technology Co., Ltd., Meyer Reagent Shanghai Co., Ltd., Macklin Reagent Shanghai Co., Ltd., Chongqing Chuandong Chemical Co., Ltd etc. without further purification unless otherwise noted. High resolution mass spectra (Guangzhou, South China Agricultural University) were measured on commercial instruments. NMR spectra were recorded on commercial instruments (Germany, Bruker company) and operated at 600 MHz for ^{1}H-NMR and 151 MHz for ^{13}C-NMR. Chemical shifts were reported in ppm from tetramethylsilane with the solvent resonance as the internal standard ($(CD_3)_2SO$, δ = 2.50, δ = 3.33) in ^{1}H-NMR spectra and chemical shifts were reported in ppm from the tetramethylsilane with the solvent resonance as internal standard ($(CD_3)_2SO$, δ = 39.5) in ^{13}C-NMR spectra. Spectra are reported as follows: chemical shift (δ ppm), multiplicity (s = singlet, d = doublet, t = triplet, q = quartet, m = multiplet), coupling constants (Hz), integration and assignment.

B. General Procedure for Halogen-metal Exchange Reaction

(5-Formyl-pyridin-2-yl)-carbamic acid tert-butyl ester (3a) [1]:

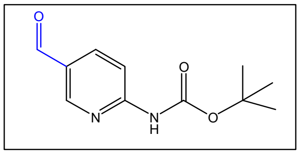

To a solution of (5-Bromo-pyridin-2-yl)-carbamic acid tert-butyl ester (1.0g, 3.7 mmol, 1.0 equiv) in dry THF (12 ml) at 0 ℃ was added a 2 M solution of *i*-PrMgCl in THF (1.85 ml, 1.0 equiv) in 5 min. The clear solution was stirred at that temperature for an additional 5 min, and a 2.5 M solution of *n*-BuLi in hexanes (3 ml, 2.0 equiv) was added dropwise in 5min ,while maintaining the temperature below -20 ℃. The resulting mixture was stirred at that temperature for 0.5h, dry DMF (0.27 g, 1.0 equiv) in dry THF (5 ml) was added dropwise in 10min.the resulting mixture was warmed to -20 ℃ in 0.5 h and added to quenched with water (6 ml). After stirring the mixture below -20 ℃ for 10 min, the phases were separated and the water phase was extracted one additional time with ethyl acetate. The resulting suspension was allowed to reach room temperature and fitered through a 0.5x1cm pad of silica gel eluting with 10ml of ethyl acetate. The filtrate was concentrated and the residue was purified by flash chromatography on silica gel (eluent: petroleum ether /ethyl acetate = 10:1) to afford product **3a** as white solid, 0.73 g (yield: 90%). ^{1}H NMR (600 MHz, DMSO) δ 10.42 (s, 1H), 9.94 (s, 1H), 8.93 – 8.60 (m, 1H), 8.17 (dd, J = 8.8, 2.3 Hz, 1H), 7.99 (d, J = 8.8 Hz, 1H), 1.48 (s, 9H). ^{13}C NMR (151 MHz, DMSO) δ 191.18, 157.08, 152.82, 152.40, 138.23, 127.30, 112.23, 80.97, 28.37.

1H-Indole-2-carboxylic acid (3b) [2]:

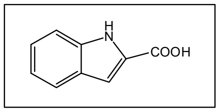

To a solution of 2-Bromo-1H-indole (1.0g, 5mmol, 1.0 equiv) in dry THF (20 ml) at 0℃ was added a 2 M solution of *i*-PrMgCl in THF (2.5 ml, 1.0 equiv) in 5 min. The clear solution was stirred at that temperature for an additional 5 min, and a 2.5 M solution of *n*-BuLi in hexanes (4 ml, 2.0 equiv) was added dropwise in 5 min ,while maintaining the temperature below -20 ℃. The resulting mixture was stirred at that temperature for 0.5h, dry CO_2 (0.22g, 1.0 equiv) was added to -20 ℃. the resulting mixture was warmed to -20 ℃ in 0.5 h and added to quenched with water (6 ml). After stirring the mixture below -20 ℃ for 10 min, the phases were separated and the water phase was extracted one additional time with ethyl acetate. The resulting suspension was allowed to reach room temperature and fitered through a 0.5 x 1cm pad of silica gel eluting with 10 ml of ethyl acetate. The filtrate was concentrated and the residue was purified by flash chromatography on silica gel (eluent: petroleum ether / ethyl acetate = 3:1) to afford product **3b** as white solid, 0.7 g (yield: 85%) , mp: 203 - 204 °C. ^{1}H NMR (600 MHz, DMSO) δ 12.94 (s, 1H), 11.76 (s, 1H), 7.79 – 6.89 (m, 5H). ^{13}C NMR (151 MHz, DMSO) δ 163.29, 137.71, 128.88, 127.34, 124.73, 122.40, 120.41, 112.95, 107.77.

1H-Indole-3-carboxylic acid (3c) [3]**：**

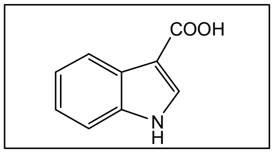

To a solution of 3-Bromo-1H-indole (0.86 g, 4.4 mmol, 1.0 equiv) in dry THF (20 ml) at 0℃ was added a 2M solution of *i*-PrMgCl in THF (2.2ml, 1equiv) in 5min. The clear solution was stirred at that temperature for an additional 5min, and a 2.5M solution of *n*BuLi in hexanes (3.5 ml, 2.0 equiv) was added dropwise in 5 min ,while maintaining the temperature below -20 ℃. The resulting mixture was stirred at that temperature for 0.5h, dry CO_2 (0.2 g, 1.0 equiv) was added to -20 ℃. The resulting mixture was warmed to -20 ℃ in 0.5 h and added to quenched with water (6 ml). After stirring the mixture below -20 ℃ for 10 min, the phases were separated and the water phase was extracted one additional time with ethyl acetate. The resulting suspension was allowed to reach room temperature and fitered through a 0.5 x 1cm pad of silica gel eluting with 10ml of ethyl acetate. The filtrate was concentrated and the residue was purified by flash chromatography on silica gel (eluent: petroleum ether / ethyl acetate = 3:1) to afford product **3c** as off-white solid, 0.63 g (yield: 89%) , mp: 193 - 196 °C. ^{1}H NMR (600 MHz, DMSO) δ 11.82 (s, 1H), 8.02 (t, J = 5.7 Hz, 2H), 7.47 (d, J = 7.6 Hz, 1H), 7.32 – 6.99 (m, 2H). ^{13}C NMR (151 MHz, DMSO) δ 166.43, 136.89, 132.71, 126.48, 122.58, 121.42, 121.05, 112.65, 107.87.

1H-Indole-5-carboxylic acid (3d) [4]:

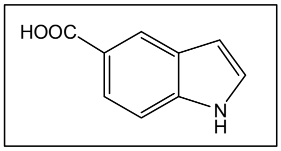

To a solution of 5-Bromo-1H-indole (0.86 g, 4.4 mmol, 1.0 equiv) in dry THF (20 ml) at 0℃ was added a 2M solution of *i*PrMgCl in THF (2.2 ml, 1.0 equiv) in 5min. The clear solution was stirred at that temperature for an additional 5min, and a 2.5 M solution of *n*-BuLi in hexanes (3.5 ml, 2.0 equiv) was added dropwise in 5.0 min ,while maintaining the temperature below -20 ℃. The resulting mixture was stirred at that temperature for 0.5h, dry CO_2 (0.2 g, 1.0 equiv) was added to -20 ℃. The resulting mixture was warmed to -20 ℃ in 0.5 h and added to quenched with water (6 ml). After stirring the mixture below -20 ℃ for 10 min, the phases were separated and the water phase was extracted one additional time with ethyl acetate. The resulting suspension was allowed to reach room temperature and fitered through a 0.5 x 1cm pad of silica gel eluting with 10 ml of ethyl acetate. The filtrate was concentrated and the residue was purified by flash chromatography on silica gel (eluent: petroleum ether / ethyl acetate = 3:1) to afford product **3d** as off-white solid, 0.46 g (yield: 65%) , mp: 210 - 214 °C. ^{1}H NMR (600 MHz, DMSO) δ 12.39 (s, 1H), 11.46 (s, 1H), 8.25 (s, 1H), 7.72 (dd, J = 8.5, 1.5 Hz, 1H), 7.45 (dd, J = 8.4, 5.7 Hz, 2H), 6.57 (s, 1H). ^{13}C NMR (151 MHz, DMSO) δ 168.90, 138.80, 127.64, 127.35, 123.28, 122.67, 121.87, 111.57, 102.93.

5-Bromo-1H-indole-3-carbaldehyde (3e) [5]**:**

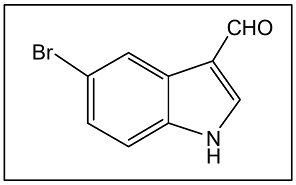

To a solution of 3,5-dibromo-1H-indole (1.2 g, 4.4 mmol, 1.0 equiv) in dry THF (20 ml) at 0 ℃ was added a 2 M solution of *i*-PrMgCl in THF (2.2 ml, 1.0 equiv) in 5 min. The clear solution was stirred at that temperature for an additional 5 min, and a 2.5 M solution of *n*-BuLi in hexanes (3.5 ml, 2.0 equiv) was added dropwise in 5 min ,while maintaining the temperature below -20 ℃. The resulting mixture was stirred at that temperature for 0.5 h, dry DMF (0.32 g, 1.0 equiv) was added to -20 ℃. The resulting mixture was warmed to -20 ℃ in 0.5 h and added to quenched with water (6 ml). After stirring the mixture below -20 ℃ for 10 min, the phases were separated and the water phase was extracted one additional time with ethyl acetate. The resulting suspension was allowed to reach room temperature and fitered through a 0.5 x 1 cm pad of silica gel eluting with 10ml of ethyl acetate. The filtrate was concentrated and the residue was purified by flash chromatography on silica gel (eluent: petroleum ether / ethyl acetate = 3:1) to afford product **3e** as yellow solid, 0.78 g (yield: 80%) , mp: 192 - 194 °C. ^{1}H NMR (600 MHz, DMSO) δ 12.35 (s, 1H), 9.93 (s, 1H), 8.47 – 8.05 (m, 2H), 7.57 – 7.21 (m, 2H). ^{13}C NMR (151 MHz, DMSO) δ 185.57, 139.67, 136.23, 126.49, 126.36, 123.39, 117.90, 115.27, 115.01.

5-Methoxy-1H-indole-2-carboxylic acid (3f) [6]:

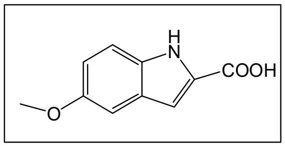

To a solution of 2-Bromo-5-methoxy-1H-indole (1.0 g, 4.4 mmol, 1.0 equiv) in dry THF (20 ml) at 0 ℃ was added a 2 M solution of *i*-PrMgCl in THF (2.2 ml, 1.0 equiv) in 5 min. The clear solution was stirred at that temperature for an additional 5 min, and a 2.5 M solution of *n*-BuLi in hexanes (3.5 ml, 2.0 equiv) was added dropwise in 5 min ,while maintaining the temperature below -20 ℃. The resulting mixture was stirred at that temperature for 0.5 h, dry CO_2 (0.20 g, 1.0 equiv) was added to -20 ℃. The resulting mixture was warmed to -20 ℃ in 0.5 h and added to quenched with water (6 ml). After stirring the mixture below -20 ℃ for 10 min, the phases were separated and the water phase was extracted one additional time with ethyl acetate. The resulting suspension was allowed to reach room temperature and fitered through a 0.5 x 1 cm pad of silica gel eluting with 10ml of ethyl acetate. The filtrate was concentrated and the residue was purified by flash chromatography on silica gel (eluent: petroleum ether / ethyl acetate = 3:1) to afford product **3f** as Brown solid, 0.68 g (yield: 80%) , mp: 199 - 201 °C. 1H NMR (600 MHz, DMSO) δ 7.36 (d, J = 8.9 Hz, 1H), 7.06 (d, J = 23.6 Hz, 2H), 6.90 (d, J = 8.8 Hz, 1H), 3.73 (s, 3H). 13C NMR (151 MHz, DMSO) δ 163.25, 154.31, 133.07, 129.10, 127.65, 116.28, 113.83, 107.47, 102.44, 55.61.

1H-Benzoimidazole-5-carboxylic acid (3g) [7]

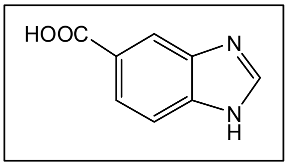

To a solution of 5-Bromo-1H-benzimidazole (0.87 g, 4.4 mmol, 1.0 equiv) in dry THF (20 ml) at 0 ℃ was added a 2 M solution of *i*-PrMgCl in THF (2.2 ml, 1.0 equiv) in 5 min. The clear solution was stirred at that temperature for an additional 5 min, and a 2.5 M solution of *n*-BuLi in hexanes (3.5 ml, 2.0 equiv) was added dropwise in 5 min ,while maintaining the temperature below -20 ℃. The resulting mixture was stirred at that temperature for 0.5 h, dry CO_2 (0.20 g, 1.0 equiv) was added to -20 ℃. The resulting mixture was warmed to -20 ℃ in 0.5 h and added to quenched with water (6 ml). After stirring the mixture below -20 ℃ for 10 min, the phases were separated and the water phase was extracted one additional time with ethyl acetate. The resulting suspension was allowed to reach room temperature and fitered through a 0.5 x 1 cm pad of silica gel eluting with 10 ml of ethyl acetate. The filtrate was concentrated and the residue was purified by flash chromatography on silica gel (eluent: petroleum ether / ethyl acetate = 3:1) to afford product **3g** as brown solid, 0.5 g (yield: 71%). ^{1}H NMR (600 MHz, DMSO) δ 8.43 (s, 1H), 8.26 (s, 1H), 7.87 (d, J = 8.4 Hz, 1H), 7.67 (d, J = 8.4 Hz, 1H). ^{13}C NMR (151 MHz, DMSO) δ 168.47, 144.83, 125.10, 123.74, 118.13, 115.12.

4-Hydroxy-benzaldehyde (3h) [8]**:**

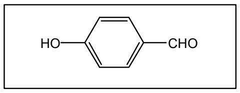

To a solution of 4-Bromo-phenol (1.5 g, 8.7 mmol, 1.0 equiv) in dry THF (25 ml) at 0 ℃ was added a 2 M solution of *i*-PrMgCl in THF (4.3 ml, 1.0 equiv) in 5 min. The clear solution was stirred at that temperature for an additional 5 min, and a 2.5 M solution of *n*-BuLi in hexanes (7.0 ml, 2.0 equiv) was added dropwise in 5 min ,while maintaining the temperature below -20 ℃. The resulting mixture was stirred at that temperature for 0.5 h, dry DMF (0.63 g, 1.0 equiv) in dry THF (5 ml) was added dropwise in 10 min. The resulting mixture was warmed to -20 ℃ in 0.5 h and added to quenched with water (6 ml). After stirring the mixture below -20 ℃ for 10 min, the phases were separated and the water phase was extracted one additional time with ethyl acetate. The resulting suspension was allowed to reach room temperature and fitered through a 0.5 x 1 cm pad of silica gel eluting with 10 ml of ethyl acetate. The filtrate was concentrated and the residue was purified by flash chromatography on silica gel (eluent: petroleum ether / ethyl acetate = 5:1) to afford product **3h** as white solid, 0.94 g (yield: 90%) ., mp: 114 - 116 °C. 1H NMR (600 MHz, DMSO) δ 10.59 (s, 1H), 9.78 (s, 1H), 7.75 (d, J = 8.6 Hz, 2H), 6.93 (d, J = 8.6 Hz, 2H). 13C NMR (151 MHz, DMSO) δ 191.35, 191.33, 163.76, 132.53, 128.89, 116.29.

N-(4-Formyl-phenyl)-acetamide (3i) [9]**:**

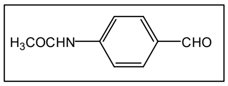

To a solution of 4-Bromo-phen N-(4-Formyl -phenyl)-acetamide (1.5 g, 7.0 mmol, 1.0 equiv) in dry THF (25 ml) at 0 ℃ was added a 2 M solution of *i*-PrMgCl in THF (3.5 ml, 1.0 equiv) in 5 min. The clear solution was stirred at that temperature for an additional 5 min, and a 2.5 M solution of *n*-BuLi in hexanes (5.6 ml, 2.0 equiv) was added dropwise in 5 min ,while maintaining the temperature below -20 ℃. The resulting mixture was stirred at that temperature for 0.5 h, dry DMF (0.5 g, 1.0equiv) in dry THF (5 ml) was added dropwise in 10min. The resulting mixture was warmed to -20 ℃ in 0.5 h and added to quenched with water (6 ml). After stirring the mixture below -20 ℃ for 10 min, the phases were separated and the water phase was extracted one additional time with ethyl acetate. The resulting suspension was allowed to reach room temperature and fitered through a 0.5 x 1cm pad of silica gel eluting with 10ml of ethyl acetate. The filtrate was concentrated and the residue was purified by flash chromatography on silica gel (eluent: petroleum ether / ethyl acetate = 10:1) to afford product **3i** as white solid, 1.0 g (yield: 94%) , mp: 157 - 158 °C. ^{1}H NMR (600 MHz, DMSO) δ 10.35 (s, 1H), 9.86 (s, 1H), 7.81 (dd, J = 32.9, 8.6 Hz, 4H), 2.10 (s, 3H). ^{13}C NMR (151 MHz, DMSO) δ 191.92, 169.55, 145.29, 131.56, 131.27, 119.01, 24.68.

1H-Pyrrole-2-carbaldehyde (3j) [10]:

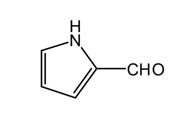

To a solution of 2-Bromo-1H-pyrrole (0.5g, 3.4 mmol, 1.0 equiv) in dry THF (15 ml) at 0 ℃ was added a 2 M solution of *i*-PrMgCl in THF (1.7 ml, 1.0 equiv) in 5min. The clear solution was stirred at that temperature for an additional 5min, and a 2.5 M solution of *n*-BuLi in hexanes (2.7 ml, 2.0 equiv) was added dropwise in 5 min ,while maintaining the temperature below -20 ℃. The resulting mixture was stirred at that temperature for 0.5 h, dry DMF (0.25 g, 1.0 equiv) in dry THF (5 ml) was added dropwise in 10min.the resulting mixture was warmed to -20 ℃ in 0.5 h and added to quenched with water (6 ml). After stirring the mixture below -20 ℃ for 10 min, the phases were separated and the water phase was extracted one additional time with ethyl acetate. The resulting suspension was allowed to reach room temperature and fitered through a 0.5 x 1 cm pad of silica gel eluting with 10ml of ethyl acetate. The filtrate was concentrated and the residue was purified by flash chromatography on silica gel (eluent: petroleum ether /ethyl acetate = 10:1) to afford product **3j** as white solid, 0.29 g (yield: 89%) , mp: 42 - 44 °C. ^{1}H NMR (600 MHz, DMSO) δ 12.19 (s, 1H), 11.69 (s, 1H), 7.04 – 6.85 (m, 1H), 6.72 (dd, J = 4.2, 2.9 Hz, 1H), 6.12 (dd, J = 5.8, 2.4 Hz, 1H). ^{13}C NMR (151 MHz, DMSO) δ 162.31, 123.82, 123.35, 115.11, 109.73.

1H-Imidazole-2-carbaldehyde (3k) [11]

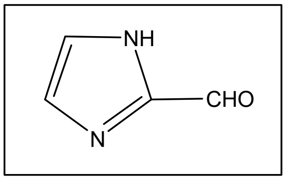

To a solution of 2-bromo-1H-imidazole (0.65 g, 4.4 mmol, 1equiv) in dry THF (20 ml) at 0℃ was added a 2 M solution of *i*-PrMgCl in THF (2.2 ml, 1.0 equiv) in 5 min. The clear solution was stirred at that temperature for an additional 5 min, and a 2.5 M solution of *n*-BuLi in hexanes (3.5 ml, 2.0 equiv) was added dropwise in 5 min ,while maintaining the temperature below -20 ℃. The resulting mixture was stirred at that temperature for 0.5 h, dry DMF (0.32 g, 1.0 equiv) was added to -20 ℃. The resulting mixture was warmed to -20 ℃ in 0.5 h and added to quenched with water (6 ml). After stirring the mixture below -20 ℃ for 10 min, the phases were separated and the water phase was extracted one additional time with ethyl acetate. The resulting suspension was allowed to reach room temperature and fitered through a 0.5 x 1 cm pad of silica gel eluting with 10 ml of ethyl acetate. The filtrate was concentrated and the residue was purified by flash chromatography on silica gel (eluent: petroleum ether / ethyl acetate = 10:1) to afford product **3k** as Pale yellow solid, 0.38 g (yield: 91%) , mp: 205 - 206 °C. ^{1}H NMR (600 MHz, DMSO) δ 13.60 (s, 1H), 9.64 (s, 1H), 7.42 (s, 2H). ^{13}C NMR (151 MHz, DMSO) δ 181.66, 146.09.

1H-Imidazole-4-carbaldehyde (3l) [12]

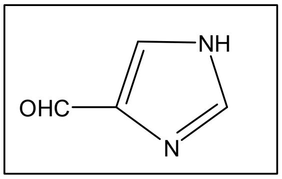

To a solution of 4-bromo-1H-imidazole (0.65 g, 4.4 mmol, 1.0 equiv) in dry THF (20 ml) at 0 ℃ was added a 2 M solution of *i*-PrMgCl in THF (2.2 ml, 1.0 equiv) in 5 min. The clear solution was stirred at that temperature for an additional 5min, and a 2.5M solution of *n*-BuLi in hexanes (3.5 ml, 2.0 equiv) was added dropwise in 5 min ,while maintaining the temperature below -20 ℃. The resulting mixture was stirred at that temperature for 0.5 h, dry DMF (0.32 g, 1.0 equiv) was added to -20 ℃. The resulting mixture was warmed to -20 ℃ in 0.5 h and added to quenched with water (6 ml). After stirring the mixture below -20 ℃ for 10 min, the phases were separated and the water phase was extracted one additional time with ethyl acetate. The resulting suspension was allowed to reach room temperature and fitered through a 0.5 x 1 cm pad of silica gel eluting with 10 ml of ethyl acetate. The filtrate was concentrated and the residue was purified by flash chromatography on silica gel (eluent: petroleum ether / ethyl acetate = 10:1) to afford product **3l** as Off-white solid, 0.36 g (yield: 85%) , mp: 175 - 177 °C. ^{1}H NMR (600 MHz, DMSO) δ 9.74 (s, 1H), 7.99 (s, 1H), 7.94(s, 1H). ^{13}C NMR (151 MHz, DMSO) δ 184.46, 139.44, 134.9, 129.5.

6-Hydroxy-pyridine-2-carboxylic acid (3m) [13]

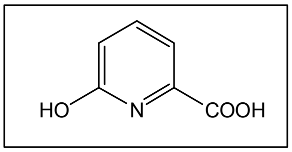

To a solution of 2-bromo-6-hydroxypyridine (0.76 g, 4.4 mmol, 1.0 equiv) in dry THF (20 ml) at 0 ℃ was added a 2 M solution of *i*-PrMgCl in THF (2.2 ml, 1.0 equiv) in 5 min. The clear solution was stirred at that temperature for an additional 5 min, and a 2.5 M solution of *n*-BuLi in hexanes (3.5 ml, 2.0 equiv) was added dropwise in 5 min ,while maintaining the temperature below -20 ℃. The resulting mixture was stirred at that temperature for 0.5 h, dry CO_2 (0.20 g, 1.0 equiv) was added to -20 ℃. The resulting mixture was warmed to -20 ℃ in 0.5 h and added to quenched with water (6 ml). After stirring the mixture below -20 ℃ for 10 min, the phases were separated and the water phase was extracted one additional time with ethyl acetate. The resulting suspension was allowed to reach room temperature and fitered through a 0.5 x 1cm pad of silica gel eluting with 10 ml of ethyl acetate. The filtrate was concentrated and the residue was purified by flash chromatography on silica gel (eluent: petroleum ether / ethyl acetate = 10:1) to afford product **3m** as Off-white solid, 0.56 g (yield: 93%) , mp: 275 - 277 °C. ^{1}H NMR (600 MHz, DMSO) δ 7.56 (dd, J = 8.9, 7.0 Hz, 1H), 6.97 (d, J = 6.8 Hz, 1H), 6.65 (d, J = 9.0 Hz, 1H). ^{13}C NMR (151 MHz, DMSO) δ 163.28, 162.67, 140.51, 137.97, 123.88, 110.42.

2-Hydroxy-nicotinic acid (3n) [14]

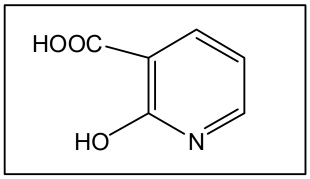

To a solution of 3-bromo-2-hydroxypyridine (0.76 g, 4.4 mmol, 1.0 equiv) in dry THF (20 ml) at 0 ℃ was added a 2 M solution of *i*-PrMgCl in THF (2.2 ml, 1.0 equiv) in 5 min. The clear solution was stirred at that temperature for an additional 5 min, and a 2.5 M solution of *n*-BuLi in hexanes (3.5 ml, 2.0 equiv) was added dropwise in 5 min ,while maintaining the temperature below -20 ℃. The resulting mixture was stirred at that temperature for 0.5 h, dry CO_2 (0.20 g, 1.0 equiv) was added to -20 ℃. The resulting mixture was warmed to -20 ℃ in 0.5 h and added to quenched with water (6 ml). After stirring the mixture below -20 ℃ for 10 min, the phases were separated and the water phase was extracted one additional time with ethyl acetate. The resulting suspension was allowed to reach room temperature and fitered through a 0.5 x 1 cm pad of silica gel eluting with 10 ml of ethyl acetate. The filtrate was concentrated and the residue was purified by flash chromatography on silica gel (eluent: petroleum ether / ethyl acetate = 10:1) to afford product **3n** as Off-white solid, 0.48 g (yield: 79%) , mp: 255 - 257 °C. ^{1}H NMR (600 MHz, DMSO) δ 14.76 (s, 1H), 13.38 (s, 1H), 8.38 (dd, J = 7.2, 2.0 Hz, 1H), 7.95 (dd, J = 6.3, 2.0 Hz, 1H), 6.68 (t, J = 6.7 Hz, 1H). ^{13}C NMR (151 MHz, DMSO) δ 165.46, 165.04, 146.61, 141.95, 117.12, 109.09.

5-Bromo-3-hydroxyethynyl-pyridin-2-ol (3o) [15]

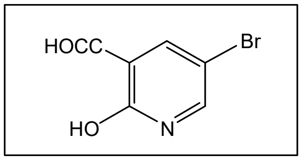

To a solution of 3,5-dibromo-2-hydroxypyridine (1.1 g, 4.4 mmol, 1.0 equiv) in dry THF (20 ml) at 0 ℃ was added a 2 M solution of *i*-PrMgCl in THF (2.2 ml, 1.0 equiv) in 5 min. The clear solution was stirred at that temperature for an additional 5 min, and a 2.5 M solution of *n*-BuLi in hexanes (3.5 ml, 2.0 equiv) was added dropwise in 5 min ,while maintaining the temperature below -20 ℃. The resulting mixture was stirred at that temperature for 0.5 h, dry CO_2 (0.20 g, 1.0 equiv) was added to -20 ℃. The resulting mixture was warmed to -20 ℃ in 0.5 h and added to quenched with water (6 ml). After stirring the mixture below -20 ℃ for 10 min, the phases were separated and the water phase was extracted one additional time with ethyl acetate. The resulting suspension was allowed to reach room temperature and fitered through a 0.5 x 1cm pad of silica gel eluting with 10 ml of ethyl acetate. The filtrate was concentrated and the residue was purified by flash chromatography on silica gel (eluent: petroleum ether / ethyl acetate = 10:1) to afford product **3o** as Off-white solid, 1.0 g (yield: 80%) , mp: 245 - 247 °C. ^{1}H NMR (600 MHz, DMSO) δ 13.82 (s, 2H), 8.31 (d, J = 2.8 Hz, 1H), 8.23 (d, J = 2.8 Hz, 1H). ^{13}C NMR (151 MHz, DMSO) δ 164.43, 163.72, 147.95, 142.51, 118.49, 99.96.

C. Copies of ^{1}H and ^{13}C NMR spectra of all products.

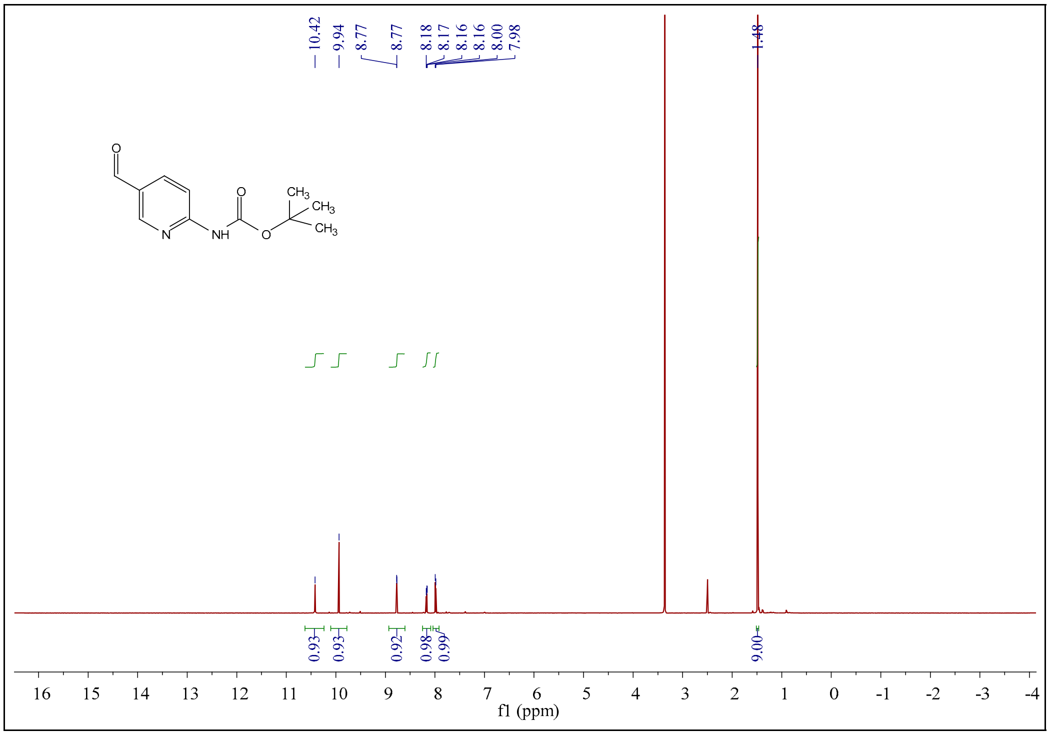

Figure 1.^{1} H NMR (600 MHz, DMSO) spectrum of **3a**.

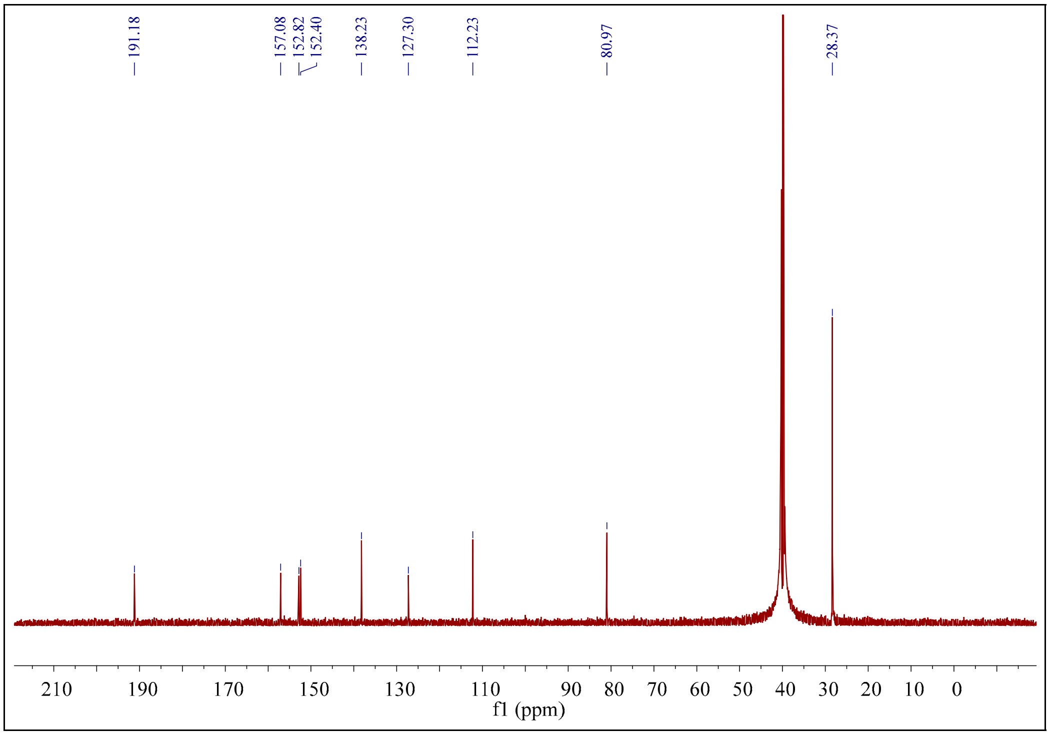

Figure 2. ^{13} C NMR (151 MHz, DMSO) spectrum of **3a.**

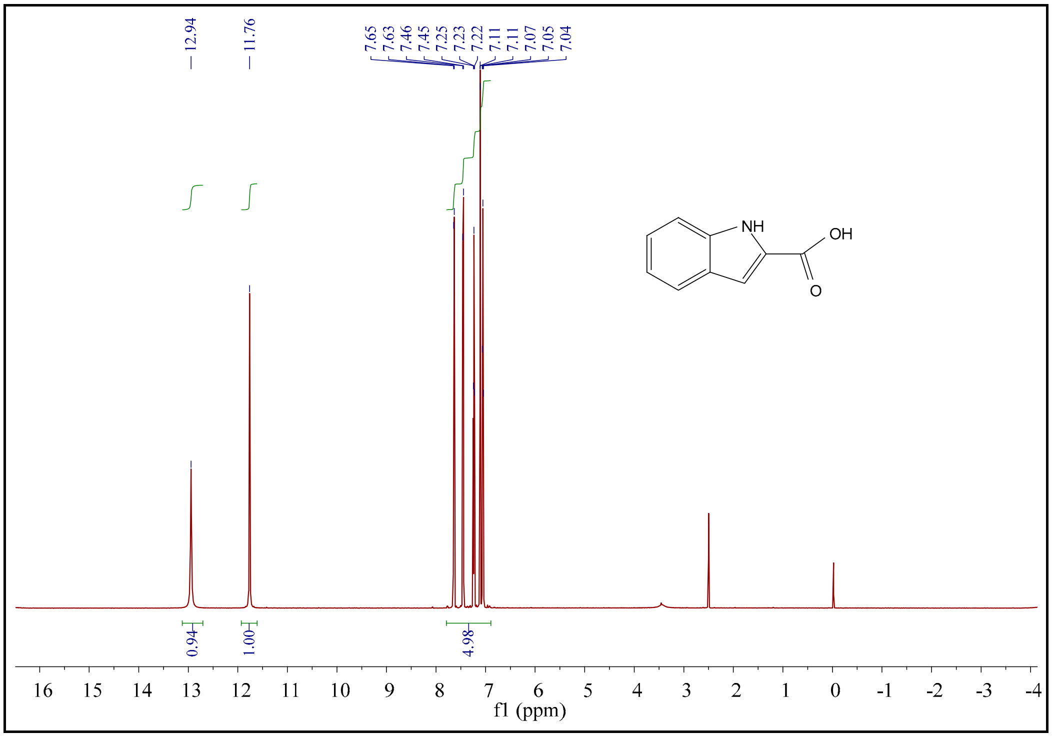

Figure 3.[1] H NMR (600 MHz, DMSO) spectrum of **3b**.

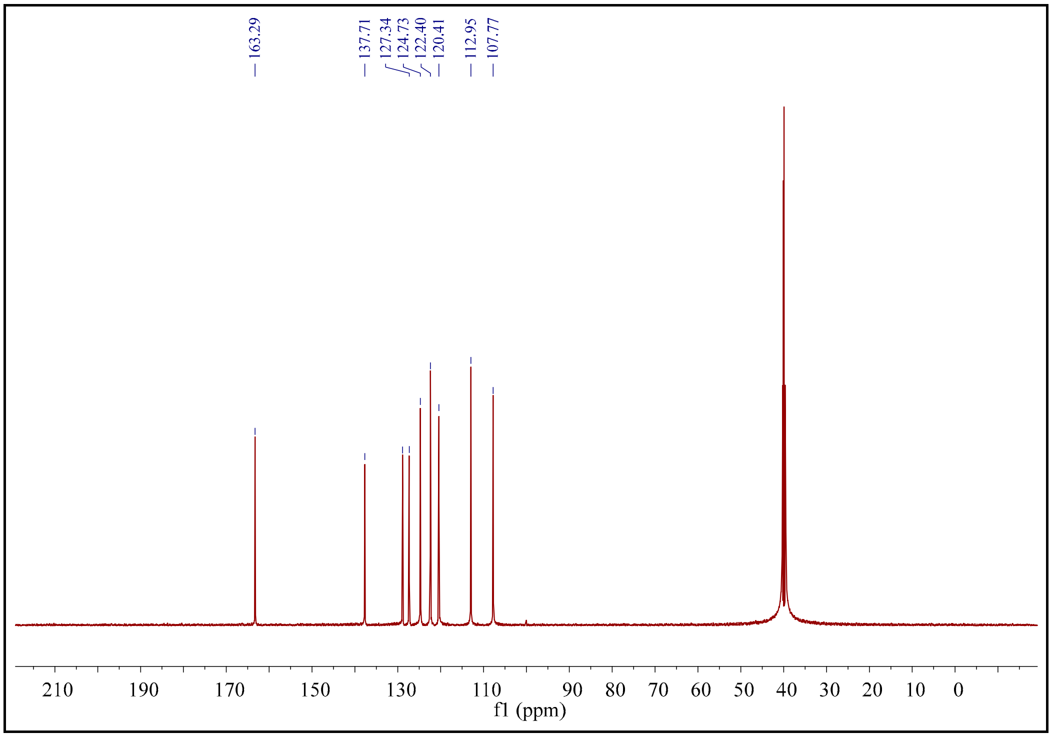

Figure 4. [13] C NMR (151 MHz, DMSO) spectrum of **3b.**

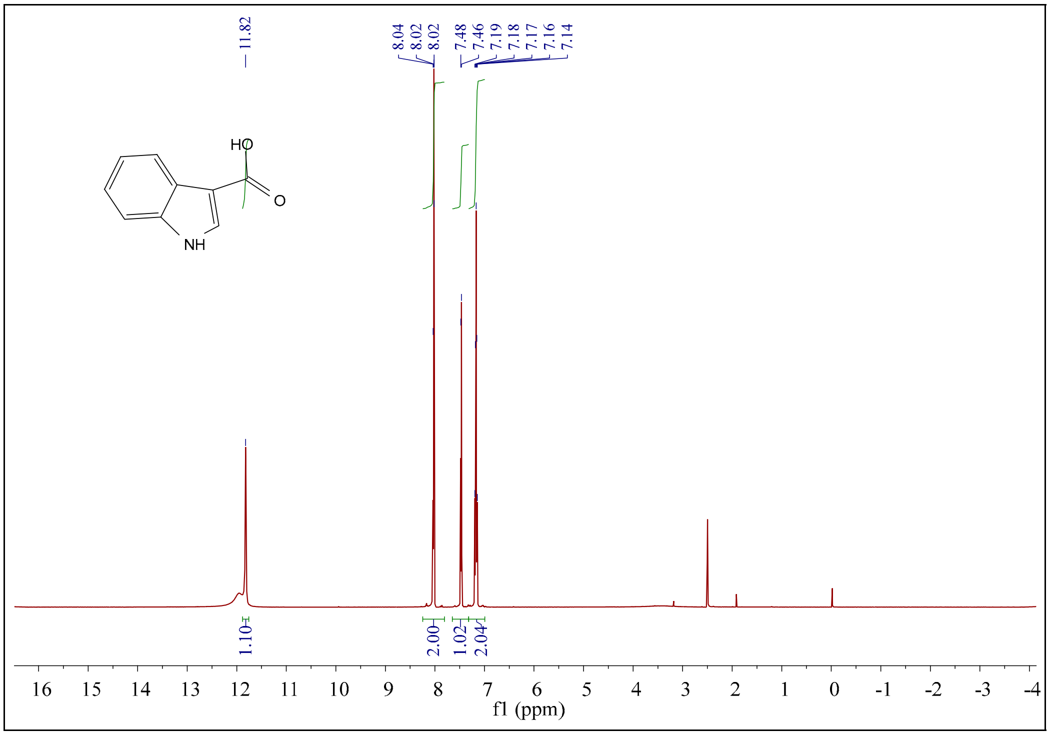

Figure 5.[1] H NMR (600 MHz, DMSO) spectrum of **3c**.

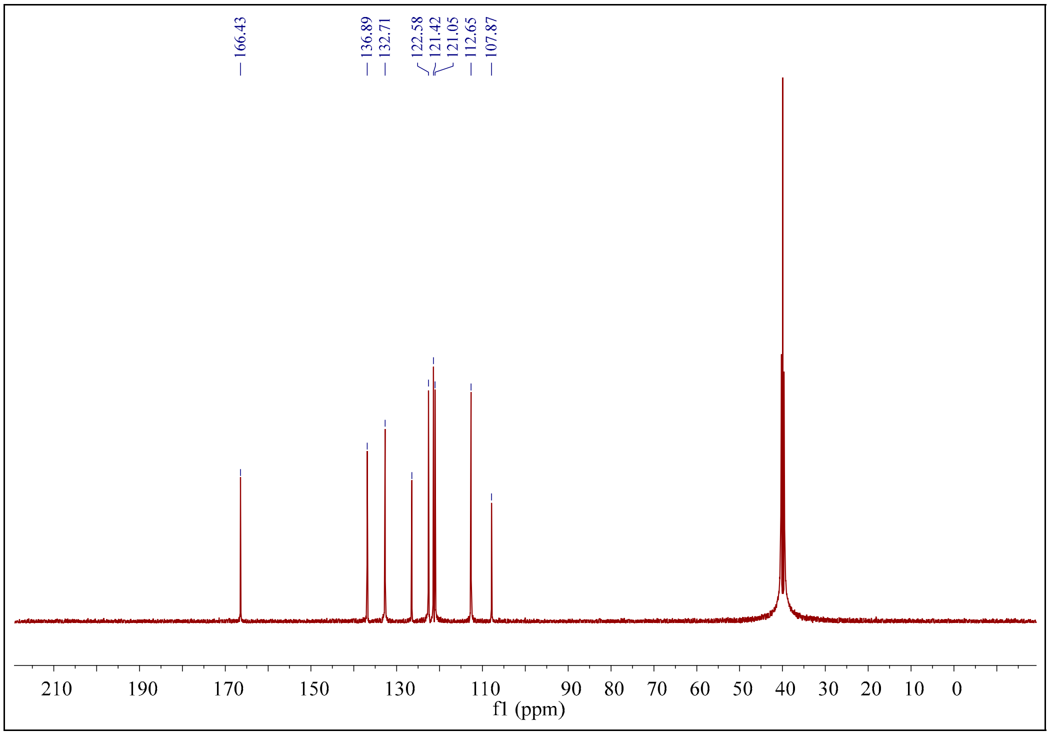

Figure 6.[1] H NMR (600 MHz, DMSO) spectrum of **3c**.

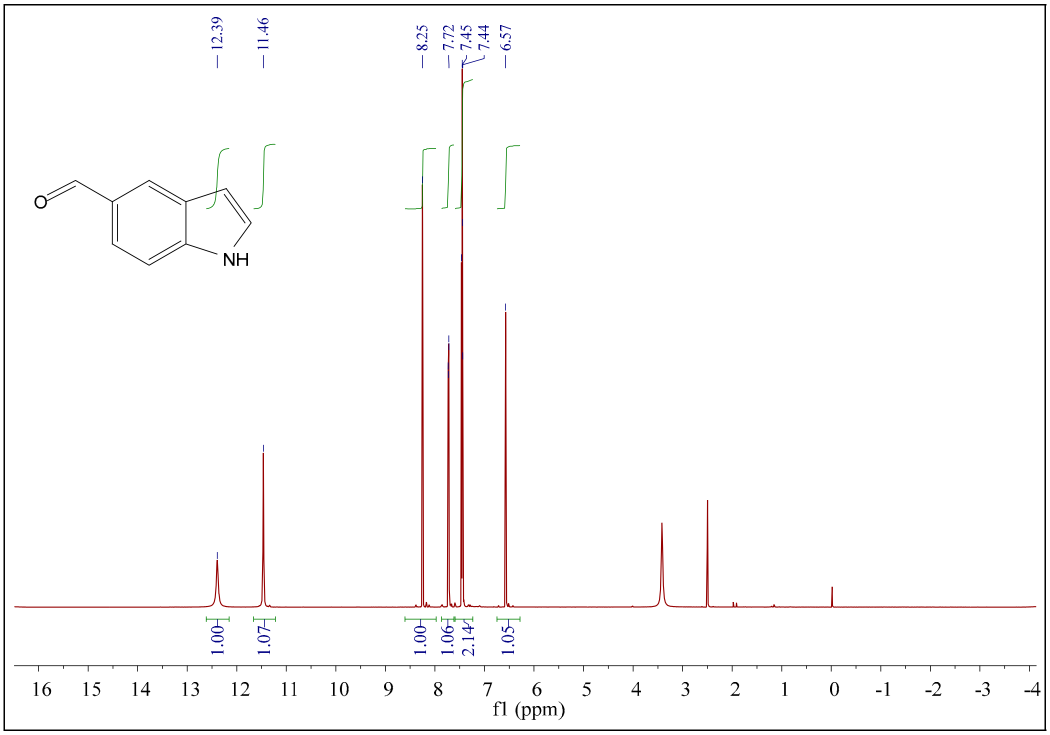

Figure 7. [1] H NMR (600 MHz, DMSO) spectrum of **3d**.

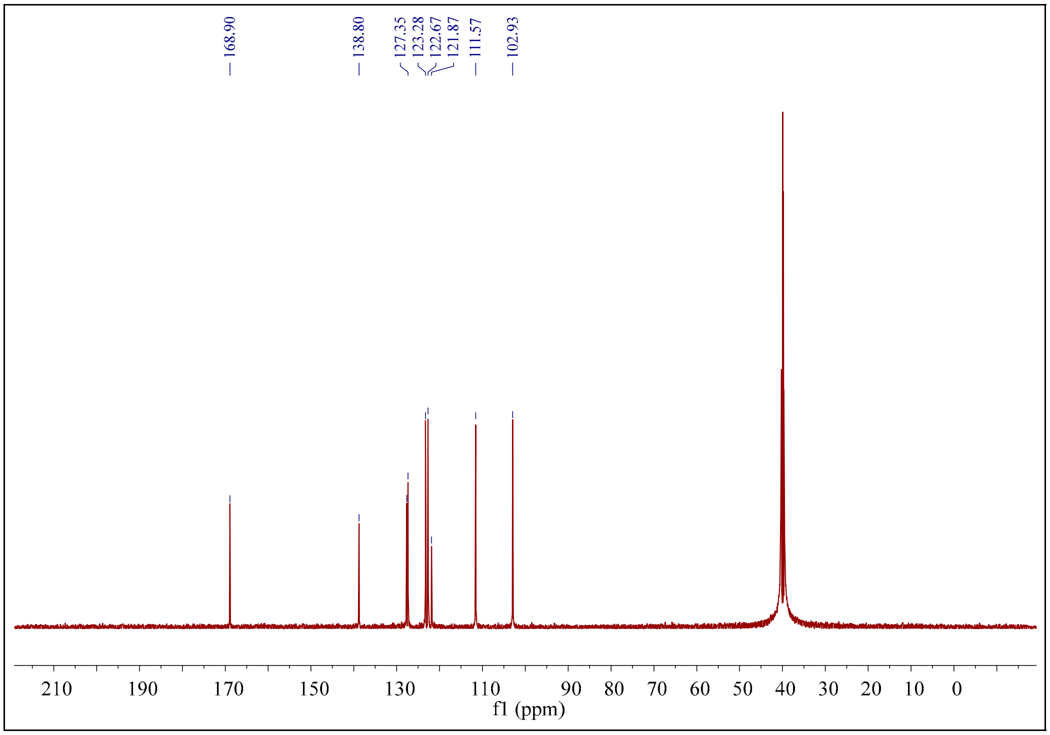

Figure 8. [13] C NMR (151 MHz, DMSO) spectrum of **3d.**

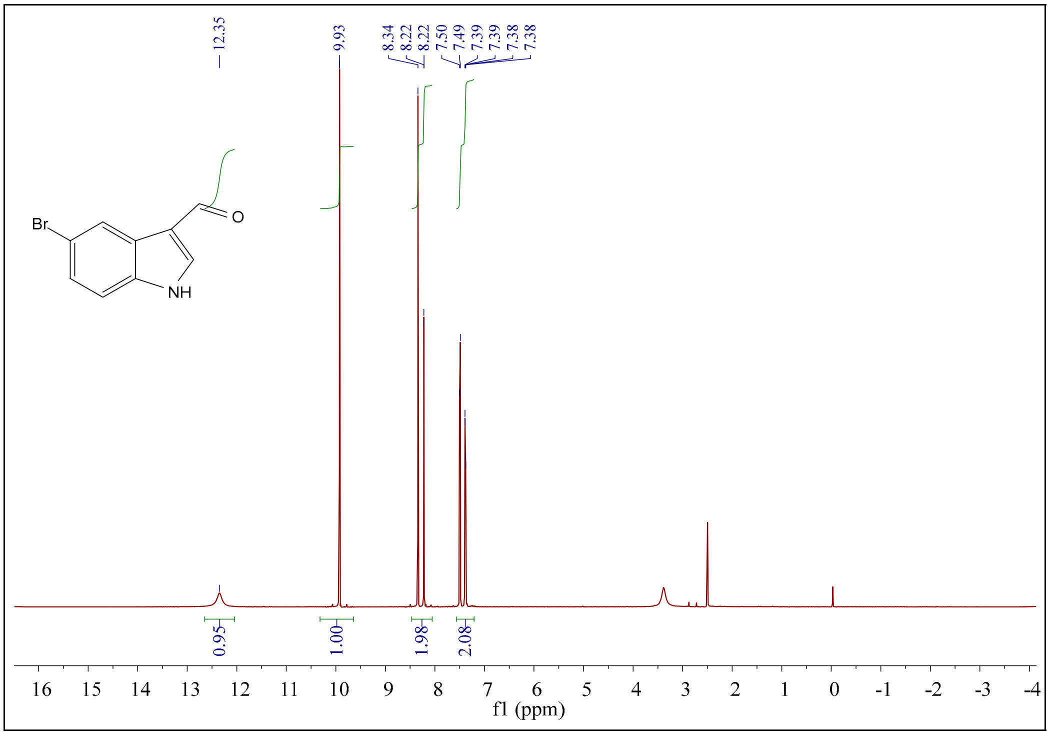

Figure 9.[1] H NMR (600 MHz, DMSO) spectrum of **3e**.

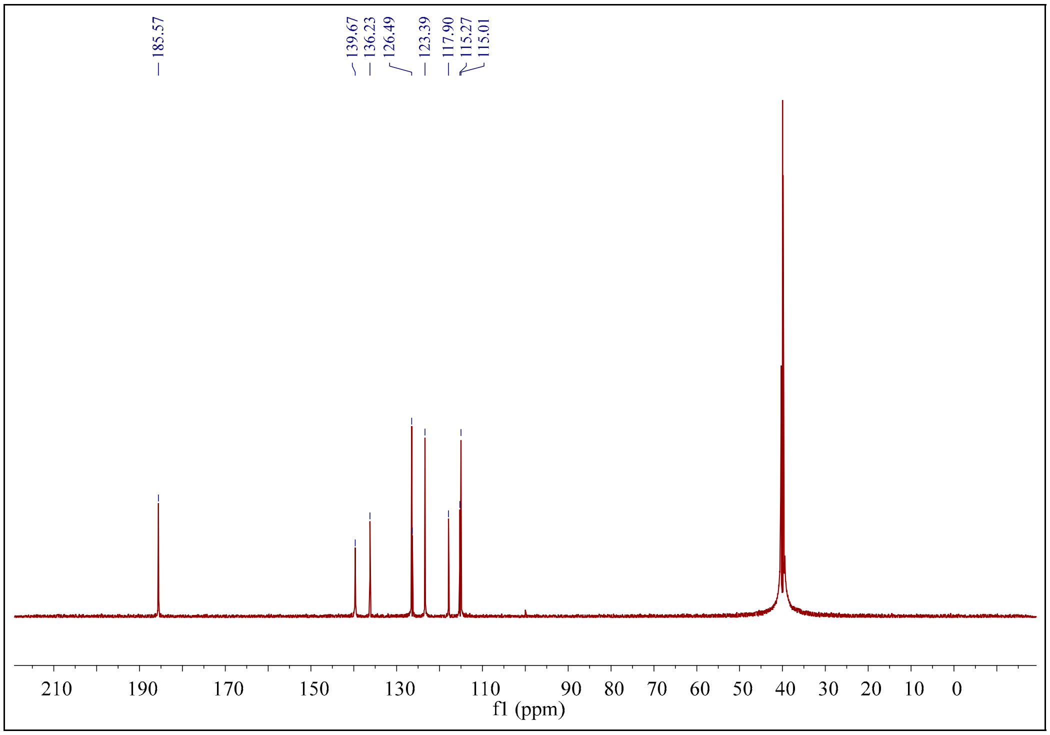

Figure 10. ^{13}C NMR (151 MHz, DMSO) spectrum of **3e**.

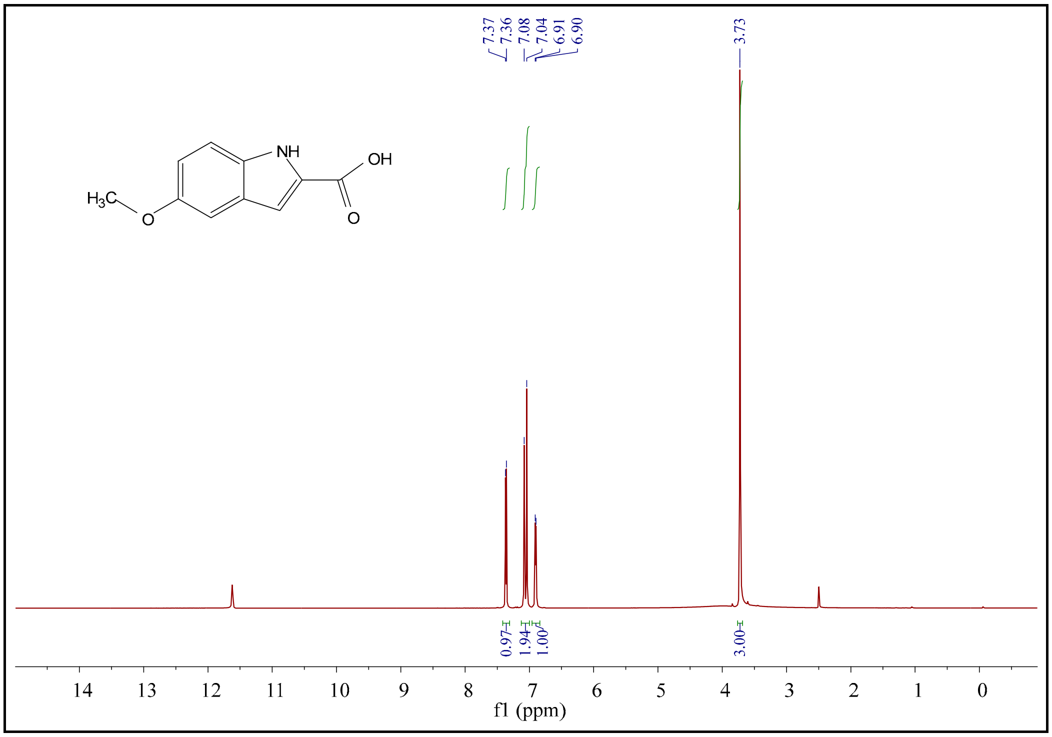

Figure 11.[1] H NMR (600 MHz, DMSO) spectrum of **3f**.

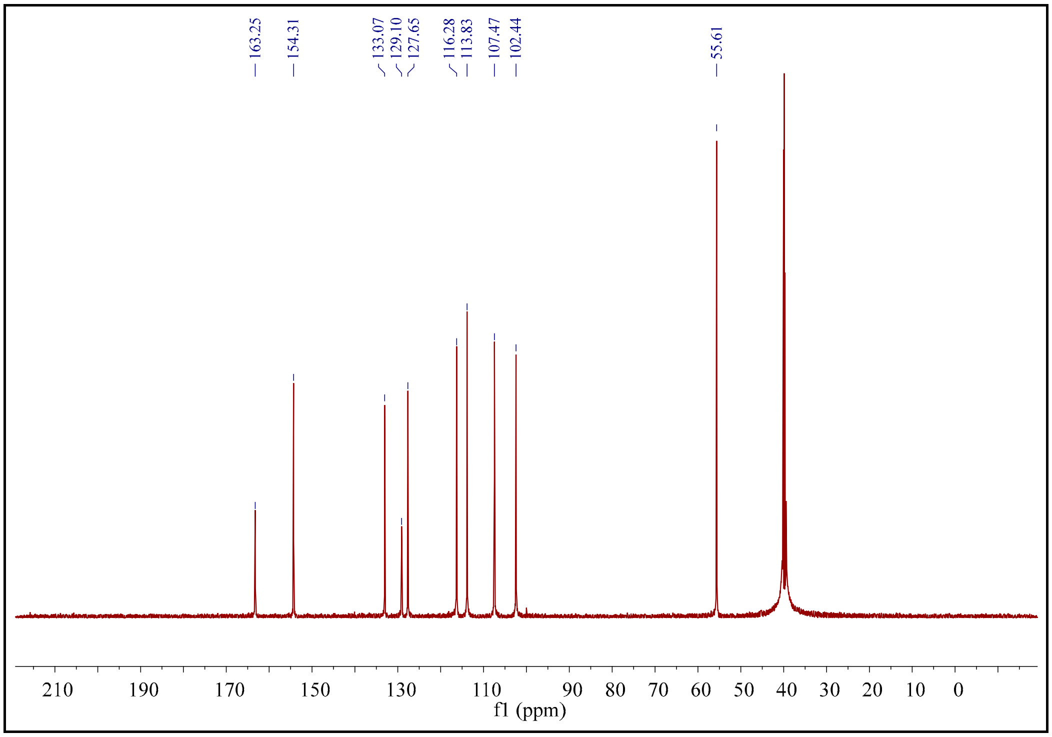

Figure 12. [13] C NMR (151 MHz, DMSO) spectrum of **3f.**

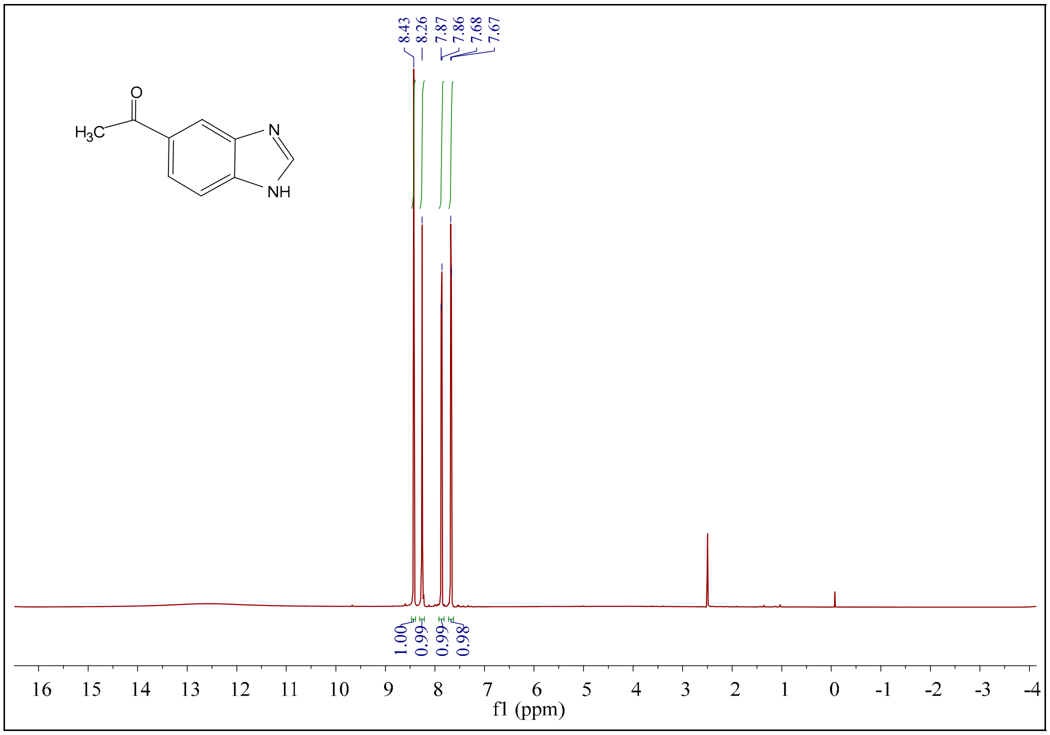

Figure 13.[1] H NMR (600 MHz, DMSO) spectrum of **3g**.

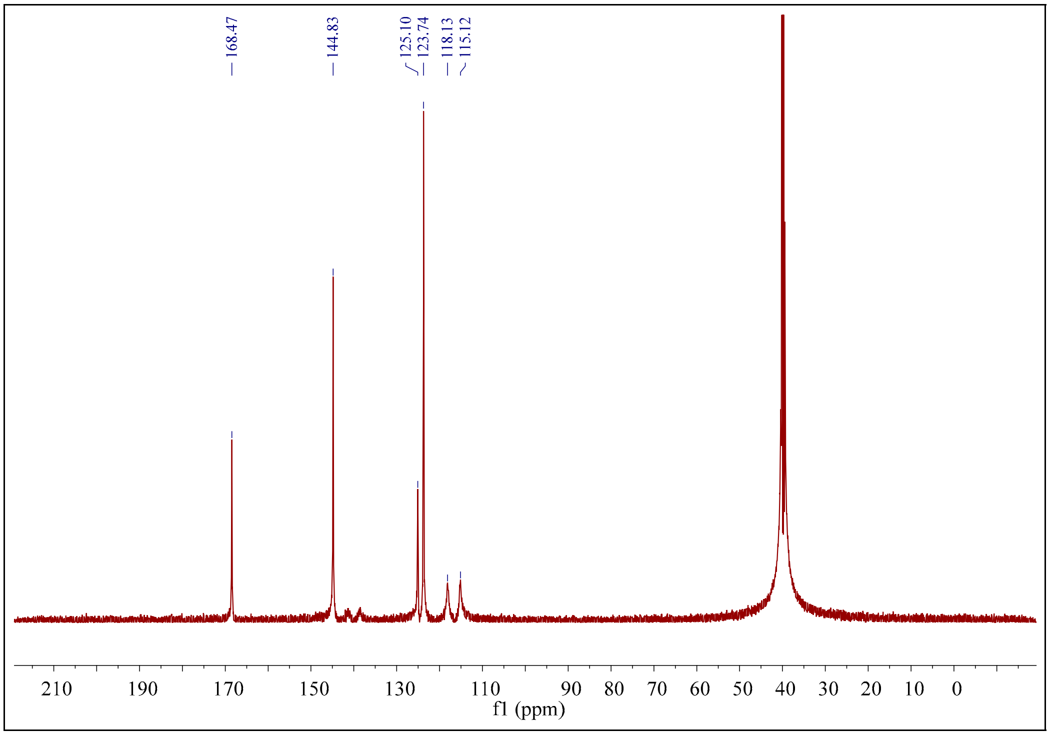

Figure 14. ^{13}C NMR (151 MHz, DMSO) spectrum of **3g.**

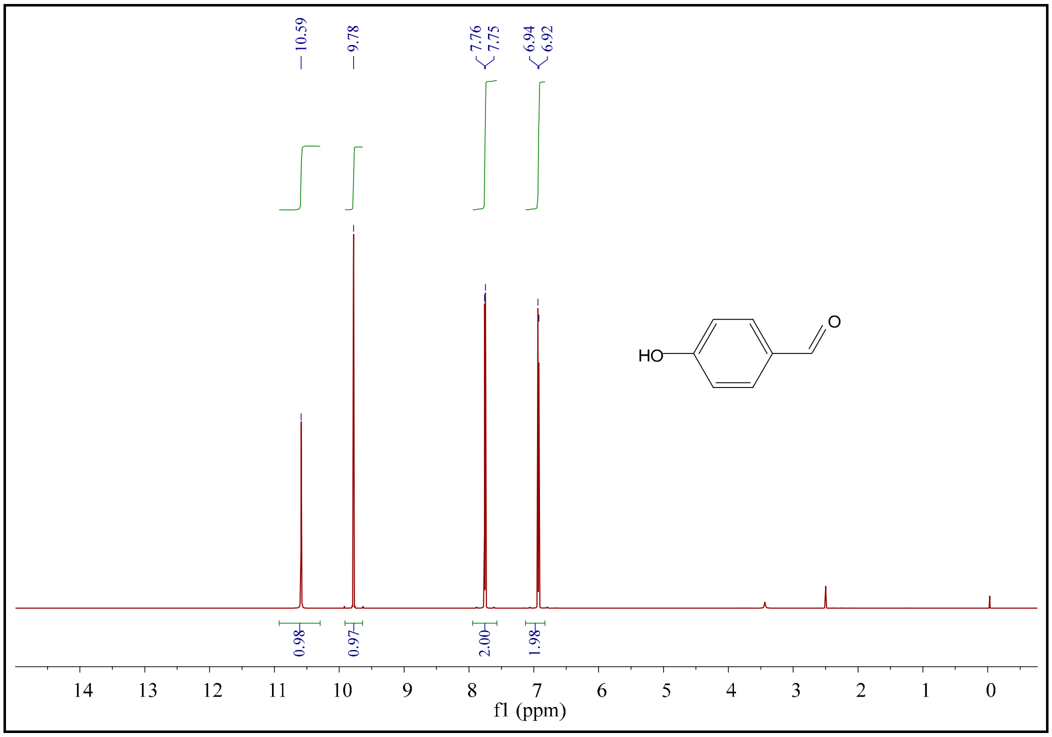

Figure 15.[1] H NMR (600 MHz, DMSO) spectrum of **3h**.

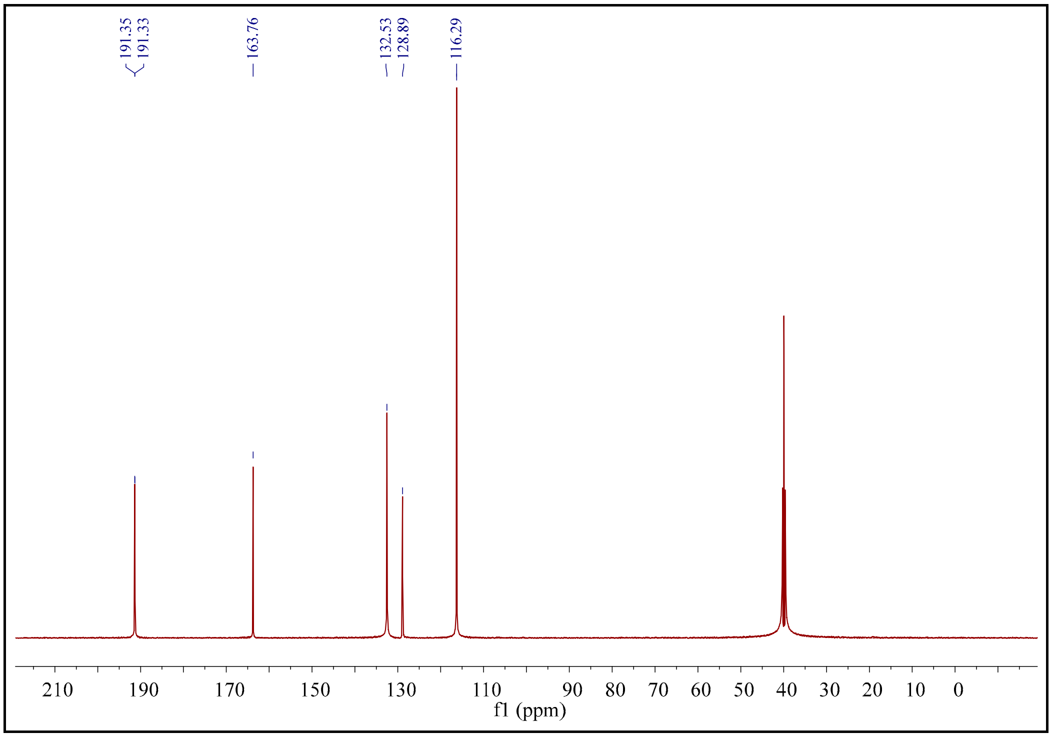

Figure 16.[13] C NMR (151 MHz, DMSO) spectrum of **3h**.

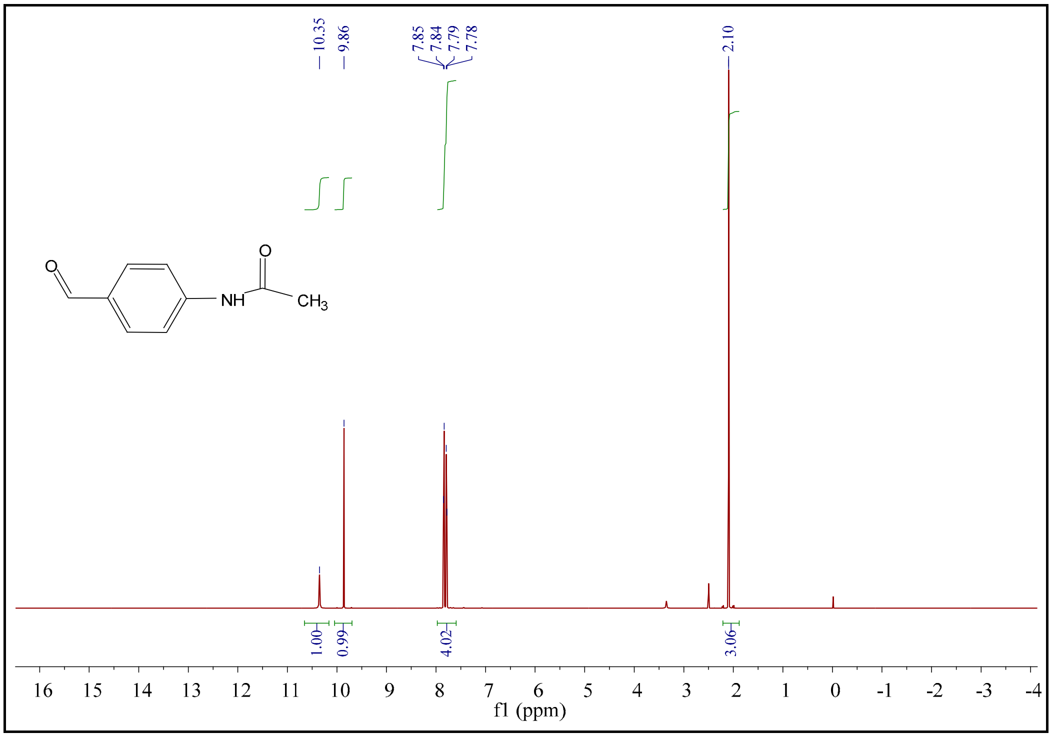

Figure 17.[1] H NMR (600 MHz, DMSO) spectrum of **3i**.

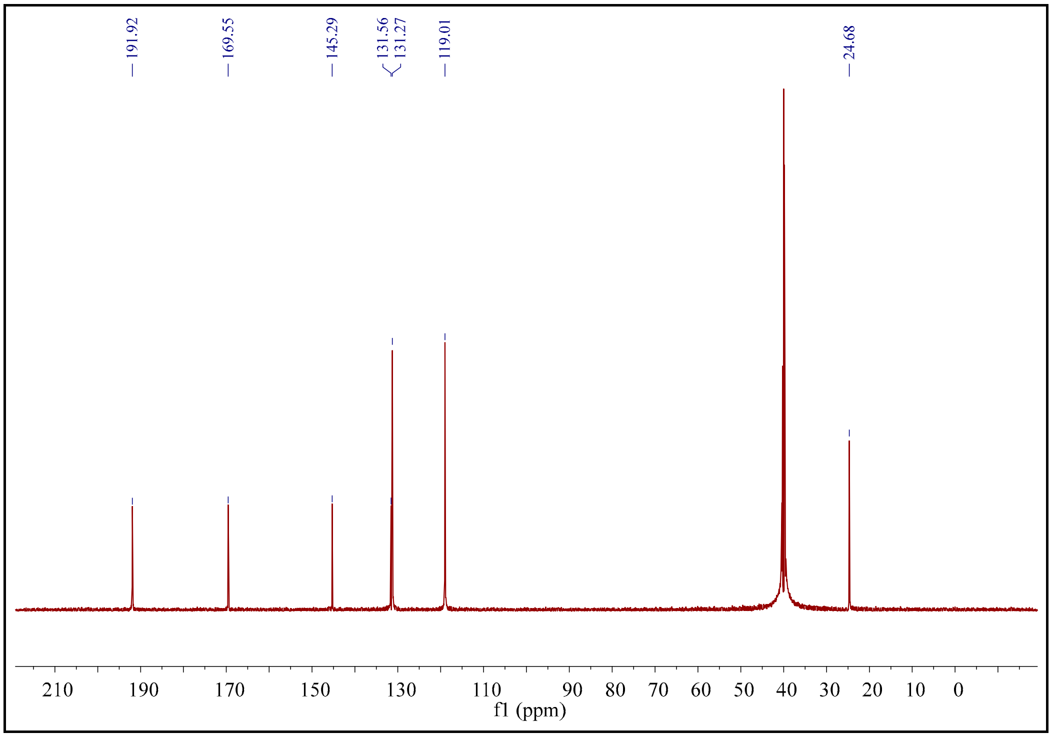

Figure 18. ^{13}C NMR (151 MHz, DMSO) spectrum of **3i.**

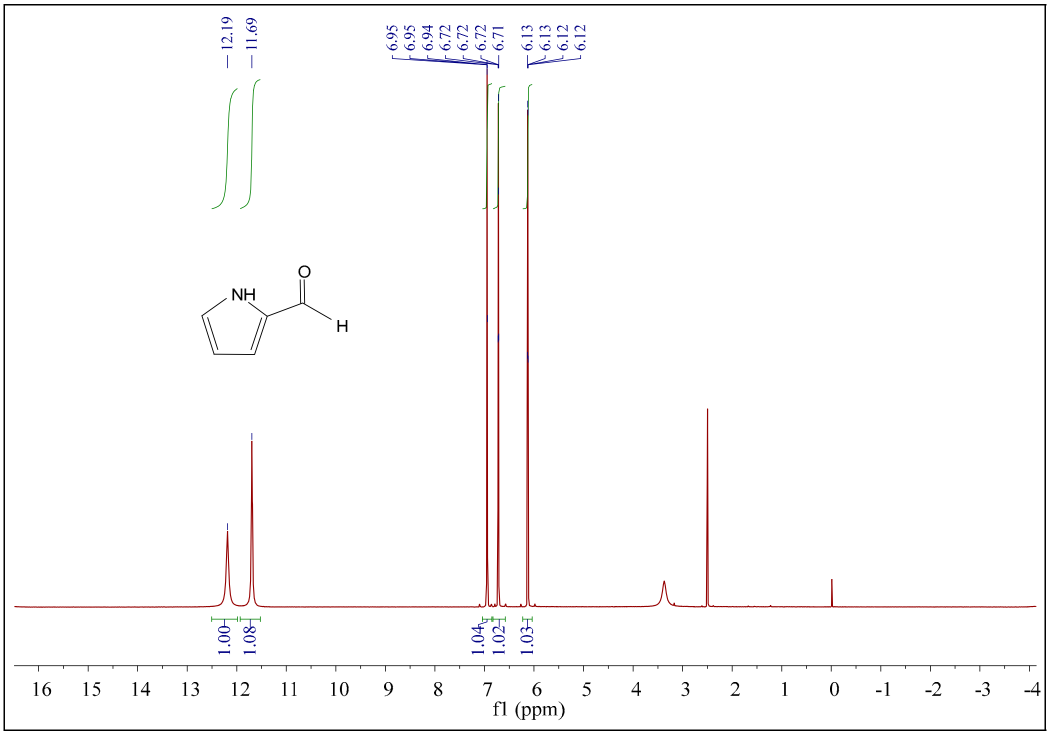

Figure 19.[1] H NMR (600 MHz, DMSO) spectrum of **3m**

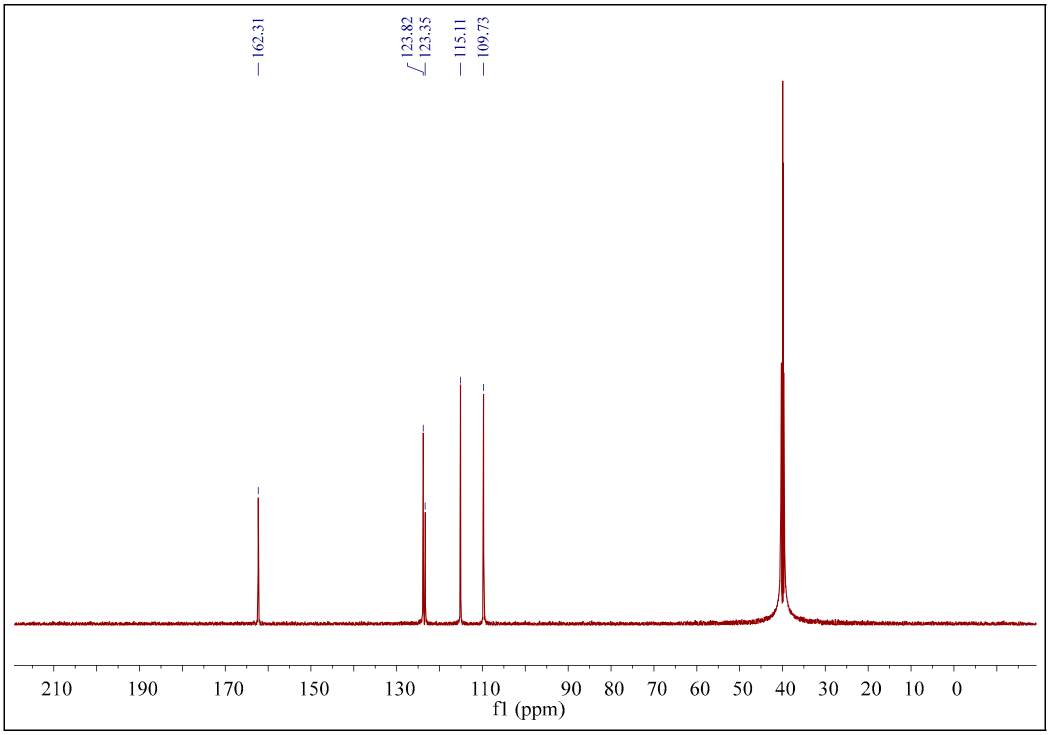

Figure 20. ^{13}C NMR (151 MHz, DMSO) spectrum of **3m.**

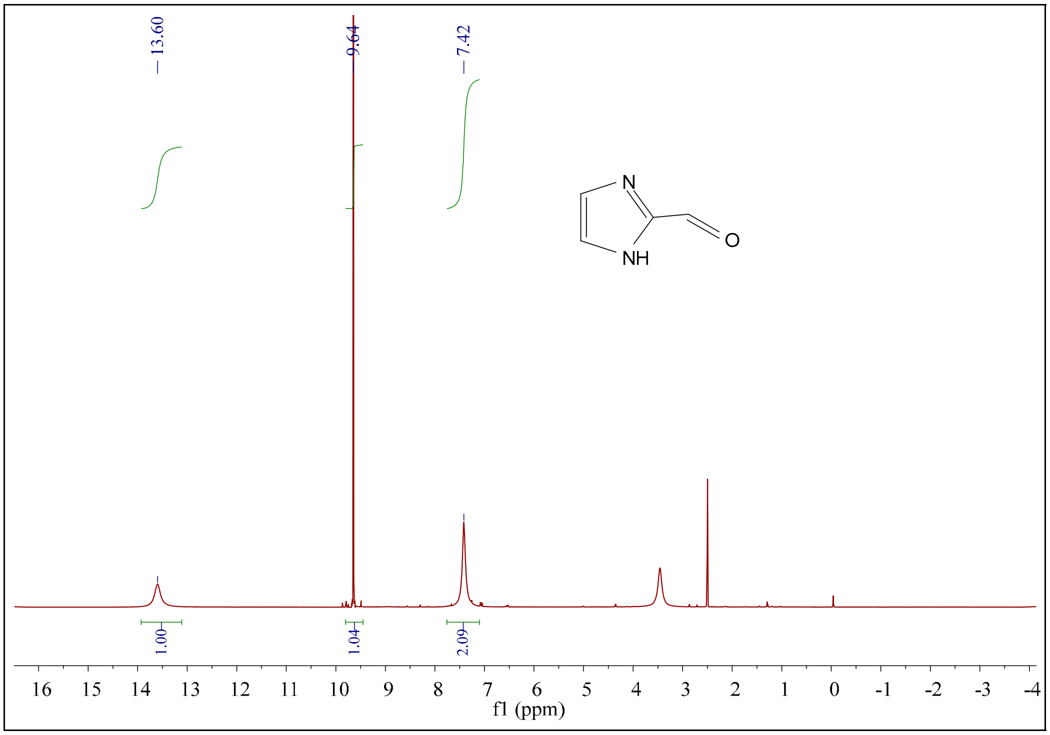

Figure 21.[1] H NMR (600 MHz, DMSO) spectrum of **3k**.

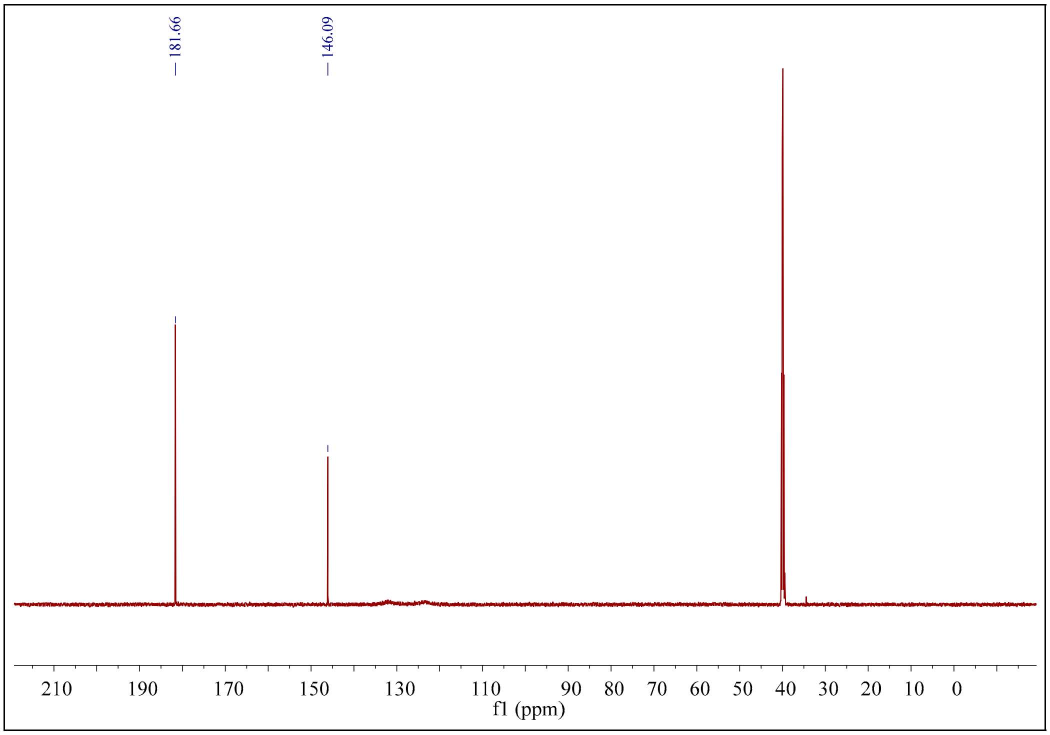

Figure 22. ^{13}C NMR (151 MHz, DMSO) spectrum of **3k.**

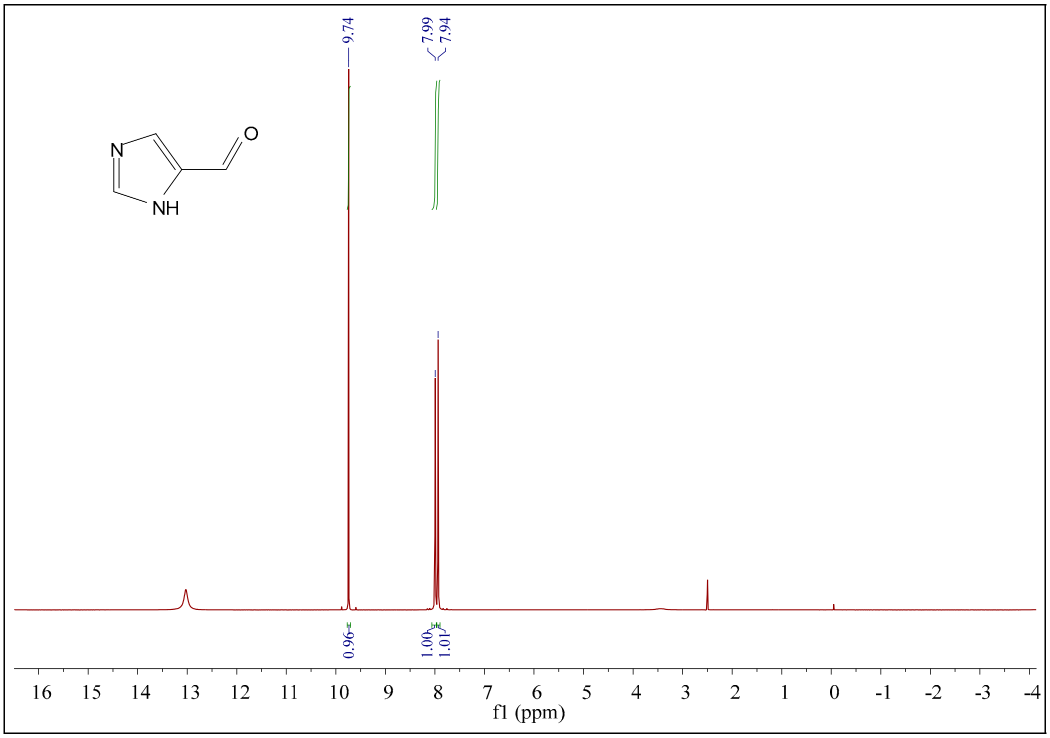

Figure 23.[1] H NMR (600 MHz, DMSO) spectrum of **3l**.

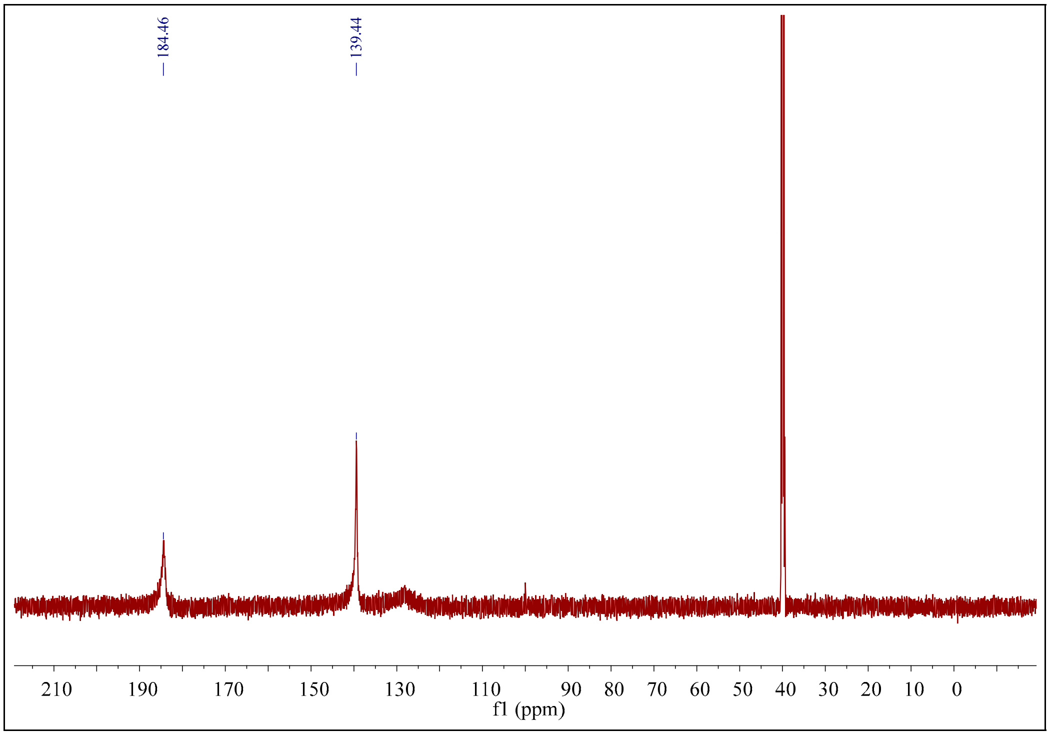

Figure 24. [13] C NMR (151 MHz, DMSO) spectrum of **3i.**

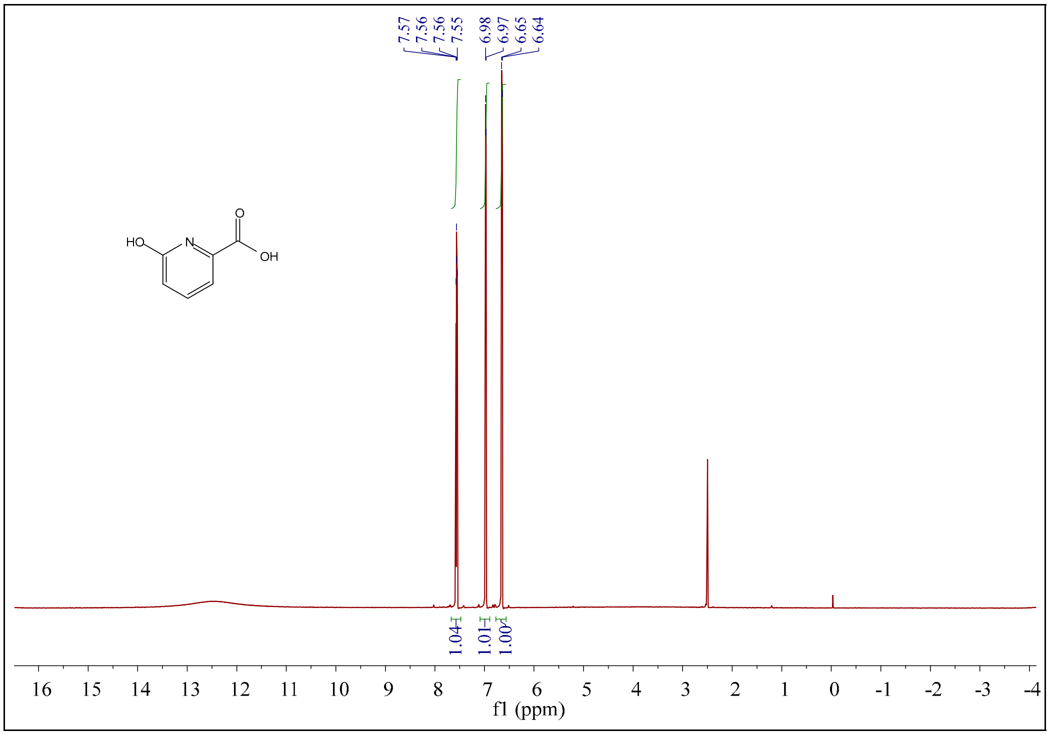

Figure 25. [1] H NMR (600 MHz, DMSO) spectrum of **3m.**

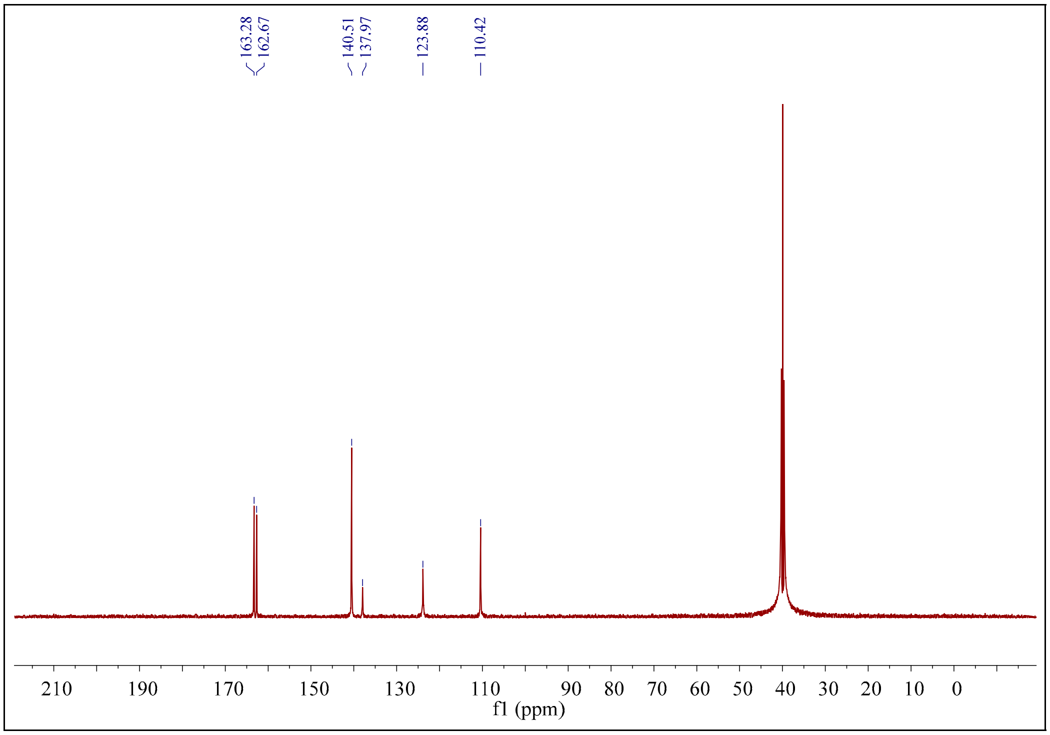

Figure 26. [13] C NMR (151 MHz, DMSO) spectrum of **3m.**

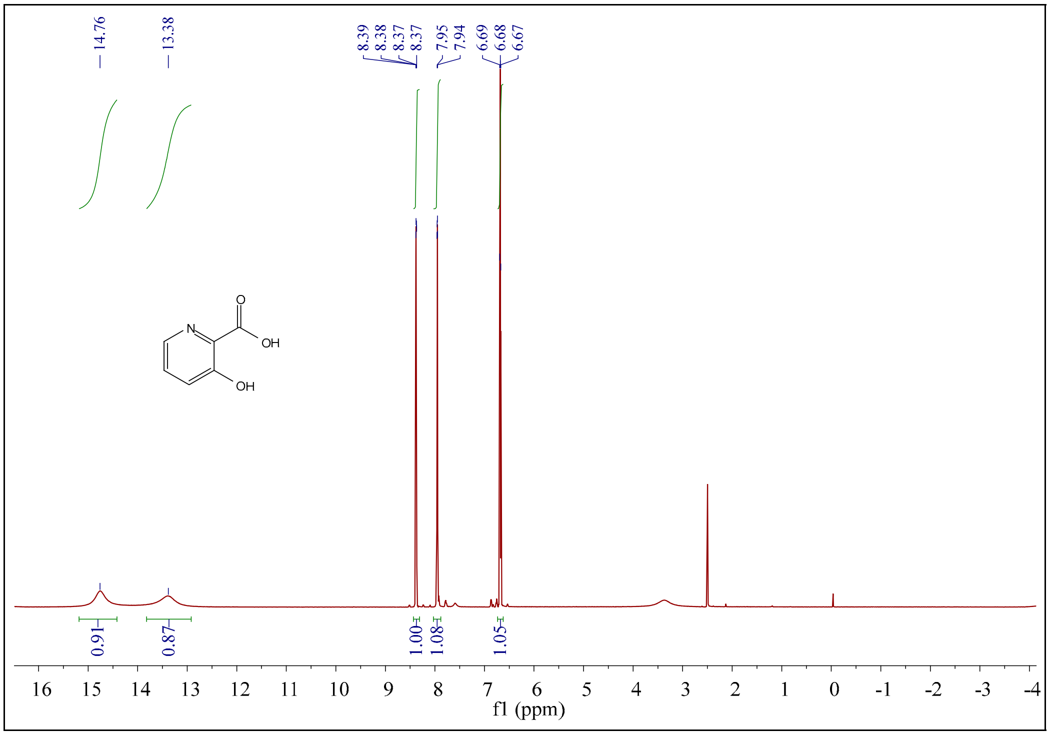

Figure 27.[1] H NMR (600 MHz, DMSO) spectrum of **3n**.

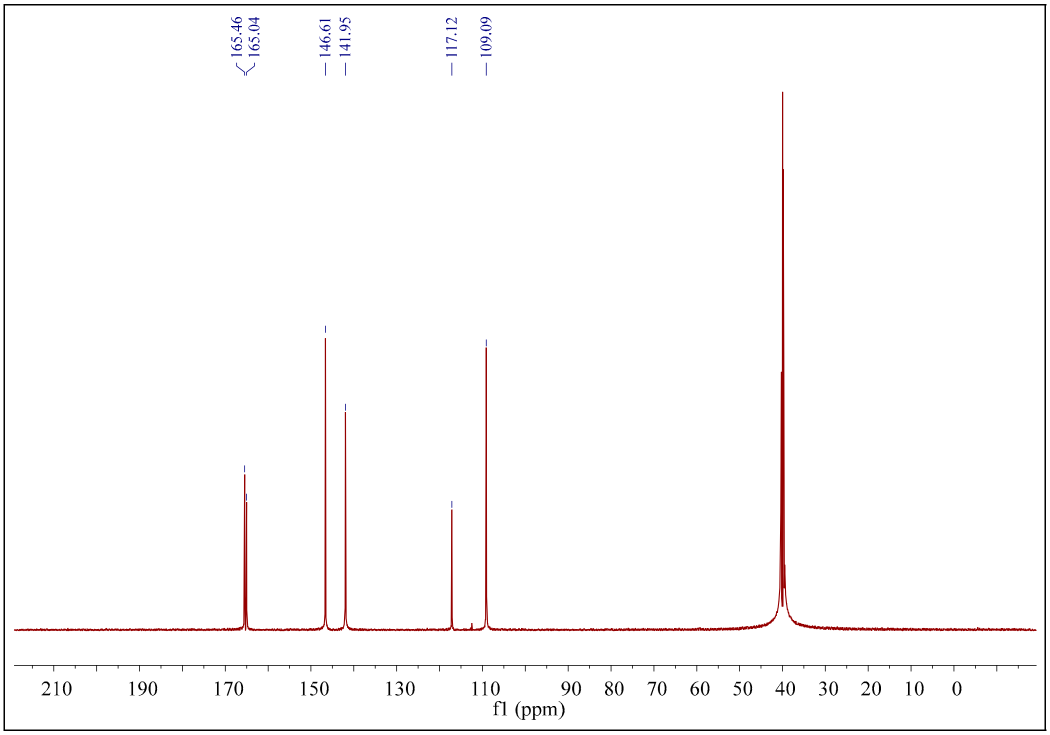

Figure 28. ^{13}C NMR (151 MHz, DMSO) spectrum of **3n.**

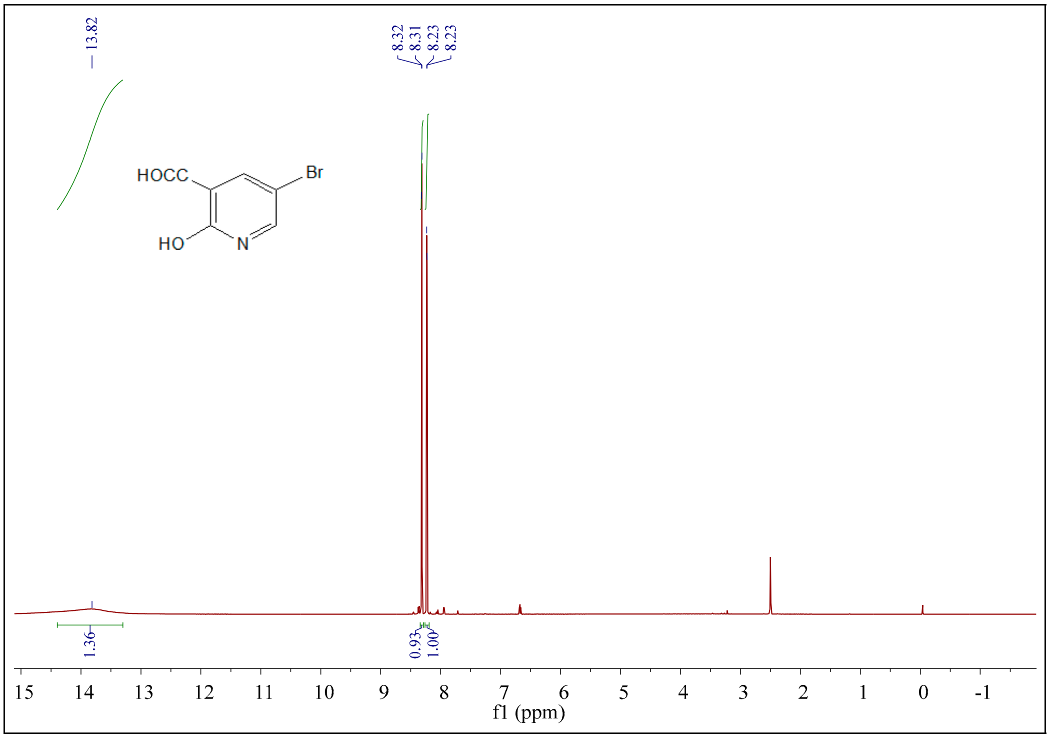

Figure 29. ^{1}H NMR (600 MHz, DMSO) spectrum of **3o.**

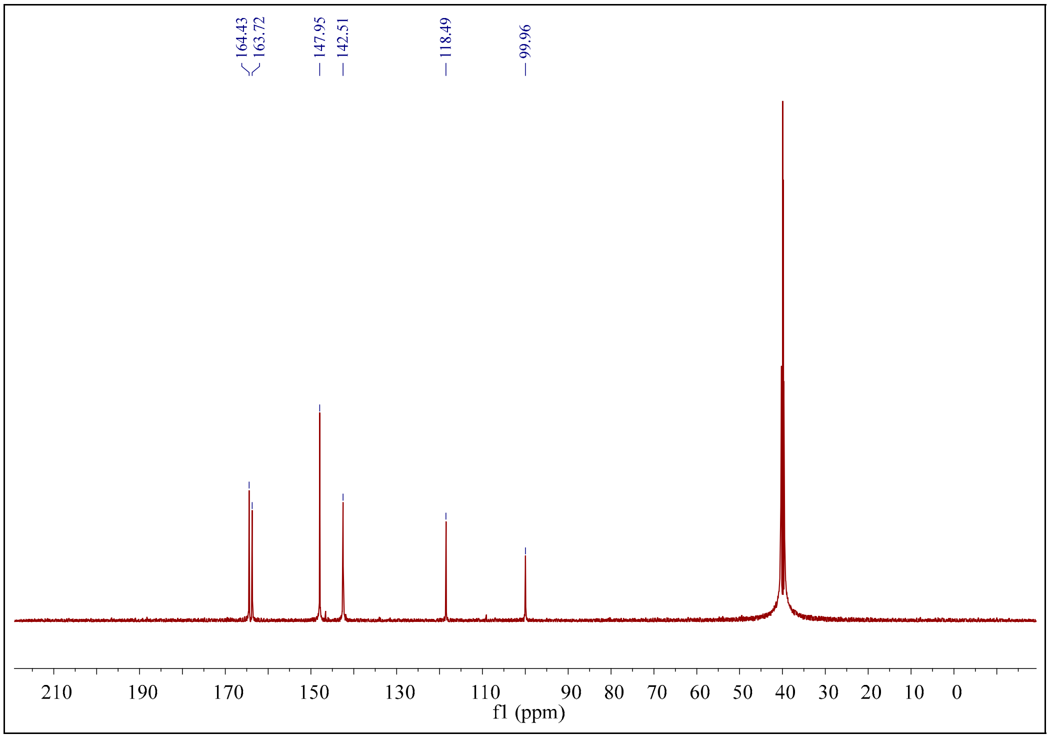

Figure 30. ^{13}C NMR (151 MHz, DMSO) spectrum of **3o.**

D. References

1 Abbvie Inc.; Michaelides, Michael; Hansen, Todd; Dai, Yujia; Zhu, Guidong; Frey, Robin; Gong, Jane; Penning, Thomas; Curtin, Michael; Mcclellan, William; Clark, Richard; Torrent, Maricel; Mastracchio, Anthony; Kesicki, Edward A.; Kluge, Arthur F.; Patane, Michael A.; Van Drie, John H. Jr.; JI, Zhiqin; Lai, Chunqiu C.; Wang, Ce Patent: WO2016/44770 A1, 2016.

2 Boraei, Ahmed T. A.; El Ashry, El Sayed H.; Barakat, Assem; Ghabbour, Hazem A. Molecules, 2016, 21, 333.

3 Wang, Xinying; Liu, Yizhou; Xu, Juan; Jiang, Fanwei; Kang, Congmin. Journal of Chemical Research, 2016 , 40, 588 – 590.

4 Gadakh, Amol V.; Chikanna, Dinesh; Rindhe, Sahebrao S.; Karale, Bhausaheb K.. Synthetic Communications, 2012, 42, 658 – 666.

5 Prajapti, Santosh Kumar; Nagarsenkar, Atulya; Guggilapu, Sravanthi Devi; Gupta, Keshav Kumar; Allakonda, Lingesh; Jeengar, Manish Kumar; Naidu; Babu, Bathini Nagendra. ioorganic and Medicinal Chemistry Letters, 2016, 26, 3024 – 3028.

6 Coowar, Djalil; Bouissac, Julien; Hanbali, Mazen; Paschaki, Marie; Mohier, Eliane; Luu, Bang. Journal of Medicinal Chemistry, 2004, 47, 6270 – 6282.

7 Arjunan; Raj, Arushma; Mythili; Mohan. Journal of Molecular Structure, 2013, 1036, 326 – 340.

8 Ilangovan, Andivelu; Anandhan, Karnambaram; Kaushik, Mahabir Prasad. Tetrahedron Letters, 2015, 56, 1081 – 1084.

9 Zhao, Hong; Chen, Qiurong; Wei, Li; Jiang, Yuanyuan; Cai, Mingzhong .Tetrahedron, 2015, 71, 27151, 8725 – 8731.

10 Li, Yu; Zhang, Yanhong; Niu, Haijun; Wang, Cheng; Qin, Chuanli; Bai, Xuduo; Wang, Wen .New Journal of Chemistry, 2016 , 40, 5245 – 5254.

11 Lazaro Martinez, Juan Manuel; Romasanta, Pablo Nicolas; Chattah, Ana Karina; Buldain, Graciela Journal of Organic Chemistry, 2010, 75, 3208 – 3213.

12 Fernandes, Rodney A.; Bethi, Venkati RSC Advances, 2014, 4, 40561 – 40568.

13 Shukla; Kaul Indian Journal of Biochemistry and Biophysics, 1973, 10, 176 – 178.

14 Sanmartin, Carmen; Dominguez, Maria Victoria; Cordeu, Lucia; Cubedo, Elena; Garcia-Foncillas, Jesus; Font, Maria; Palop, Juan Antonio Archiv der Pharmazie, 2008, 341, 28 – 41.

15 Cale Jr.; Gero; Walker; Lo; Welstead Jr.; Jaques; Johnson; Leonard; Nolan Journal of Medicinal Chemistry, 1989, 32, 2178 – 2199.
